# Supplementary material for: Gender integration and female participation in scientific and health research in Zambia: a descriptive cross-sectional study protocol
Source: BMJ Open. 2023 Mar 6;13(3):e064139. doi: 10.1136/bmjopen-2022-064139 (PMC9990657; doi:10.1136/bmjopen-2022-064139)
Supplement: Supplementary data [file bmjopen-2022-064139supp001.pdf]

Table 1: IDI participant distribution per institution

Participant	UNZA	CBU	Mulungushi	Nkrumah	TDRC	Mt Makulu	Total
Registrar	1	1	0	0	0	0	2
Head of institution	0	0	0	0	1	1	2
Administrative staff	2	2	2	2	0	0	8
Technical staff	0	0	0	2	2	2	4
Lecturers	4	4	2	2	0	0	14
Total	7	7	4	6	3	3	30
