# Supplementary material for: Gender integration and female participation in scientific and health research in Zambia: a descriptive cross-sectional study protocol
Source: BMJ Open. 2023 Mar 6;13(3):e064139. doi: 10.1136/bmjopen-2022-064139 (PMC9990657; doi:10.1136/bmjopen-2022-064139)
Supplement: Supplementary data [file bmjopen-2022-064139supp002.pdf]

QUESTIONNAIRE FOR ACADEMICIANS & RESEARCHERS

Exploring the gender dimensions and factors affecting female participation in Science and Health Research in Zambia

QUESTIONNAIRE ID…………………………………………………………………………………….

NAME OF INSTITUTION: ……………………………………………………………………………………

SCHOOL: ……………………………………………………………………………………………

DEPARTMENT: ……………………………………………………………………………………….

Summary of the study

Despite the Zambian government making remarkable progress on prioritizing gender mainstreaming, female participation in science, technology and innovation (STI) in academia, research and development is still low. Furthermore, information on the integration of gender dimension and the factors affecting inclusion of men and women in STI in research & development is scant. The aim of this research project is to determine the integration of gender dimensions and the factors that influence female participation in scientific and health research in the country in order to bridge the knowledge gaps and inform policy and programming to support the advancement of gender equality in STI in the country. To this end, the National Science Technology Council has funded this study.

Instructions

You are one of the 400 participants requested to take this questionnaire. Please read carefully and answer all the questions. This questionnaire (60 questions) seeks to collect information on your opinion and perspectives on the extent to which females are involved in and the factors influencing female participation in science-related fields of study in Zambia.

Sialubanje C, *et al. BMJ Open* 2023; 13:e064139. doi: 10.1136/bmjopen-2022-064139

Section A: Demographic Characteristics

1. Age in years: ………………………………………….

2. Sex:
    1. Male
    2. Female

3. Marital status:
    1. Single
    2. Married
    3. Divorced
    4. Widow
    5. Cohabiting

4. If married, is your partner currently employed?
    1. Yes
    2. No
    3. Not applicable (select this option if not married)

5. If the answer is yes, is your partner working in this organization?
    1. Yes
    2. No

6. Do you have children?
    1. Yes
    2. No

7. If yes, indicate how many (actual number): …………………….

8. Do you have dependants (children or other persons under your responsibility)?
    1. Yes
    2. No

9. If yes, how many dependants are under your care? Indicate actual number: ……………..

10. Age of dependants:
    1. ___________ between 0 and 5 years
    2. ___________ between 5 and 18 years
    3. ___________ between 19 and 65 years
    4. ___________ 65 years and over

Section B : Educational and Career

11. Indicate your highest academic qualification

    1. Diploma
    2. BSc/BA
    3. MSc/MA
    4. PhD
    5. Postdoc

12. Field of Study (select one most appropriate):
    1. Natural Sciences
    2. Mining
    3. Engineering
    4. Medicine
    5. Veterinary Medicine
    6. Public Health
    7. Health Sciences
    8. Nursing
    9. Others (specify) :………………….

13. Did anyone influence your choice of career?
    1. Yes
    2. No

Sialubanje C, *et al. BMJ Open* 2023; 13:e064139. doi: 10.1136/bmjopen-2022-064139

14. If yes, who influenced your choice of the career
    1. Personal interest
    2. Father
    3. Mother
    4. Sibling
    5. Family member
    6. Friend
    7. Teacher
    8. Career counsellor
    9. Others (specify)……………………………………………..

15. Why did you enroll in this program initially?
    1. Interest in the subject matter
    2. Field in which I excelled
    3. Influence of faculty members
    4. Influence of advisor or mentor
    5. Prospects for personal development
    6. Social aspect of expected job
    7. Work conditions and benefits expected
    8. I always thought I would study in this field
    9. Other, please specify:……………….

Section C: Employment

16. Employment status:
    1. Permanent and Pensionable
    2. Contractual

17. Indicate the number of years working in this institution…..……….

18. Indicate your current position:
    1. Staff development fellow;

2. Lecturer 1
3. Lecturer 2
4. Lecturer 3
5. Associate Professor Researcher
6. Full Professor
7. Administrative staff

19. Indicate the number of years in this position…………………..

20. From your personal point of view, which one of the following factors were important in obtaining your current position?
    1. Earlier cooperation with faculty members of your current work place
    2. Personal contacts at your current work place previous to obtaining your position
    3. A network of people who could testify to your professional qualifications
    4. Programs promoting gender parity at work
    5. Expansion of higher education sector
    6. Formal or informal professional experience outside the academic sector
    7. Number of publications or patents
    8. Number of grants awarded
    9. Citation impact of prior work
    10. Value of grants awarded

21. To what extent are you satisfied by your current job? Please rate your satisfaction using the scale below

1	2	3	4	5
Very unsatisfactory	Unsatisfactory	I don't know	Satisfactory	Very satisfactory

Section B: Academic Research History

22. Have you been involved in academic research?

1. Yes
2. No

If the answer is No, indicate "Not applicable" on questions 20 to 22, and proceed to question 23.

23. If the answer is yes, at what level have you been involved?
    1. Research Assistant
    2. Field Supervisor
    3. Collaborator
    4. Co-investigator
    5. Principal investigator
    6. Not applicable

24. If the answer is yes, indicate number of research projects in past 2 years:
    1. 1-2
    2. 2-3
    3. 3-4
    4. 5-6
    5. >6
    6. Not applicable

25. Indicate reasons for involvement in research:
    1. Requirement for certification
    2. Academic promotion
    3. Contribution to knowledge
    4. For international recognition
    5. Not applicable
    6. Other (specify)………

26. If the answer is no, indicate the reason:
    1. Limited skills in research;
    2. Limited access to research funds;
    3. Restrictive work policy;

4. Not interested in research;
5. Other (specify)………..

27. Are you currently seeking a new job?
    1. Yes
    2. No

28. If yes, why?
    1. Better salary
    2. Closer to family
    3. Better work environment
    4. More suitable job to my training & experience
    5. Other (specify)……………..

29. Is this your first job in your field?
    1. Yes
    2. No

30. Do you consider yourself to have enough opportunities to participate in research projects?
    1. Yes
    2. No

31. If yes, mention which ones
    1. Locally funded research projects
    2. Internationally funded research projects
    3. Both locally and internationally funded research projects
    4. Not applicable

32. If the answer is no, mention why?
    1. Did not receive any funding
    2. Did not receive any information on funding opportunities
    3. Could not find information on funding opportunities
    4. Was not authorized to apply for research funds
    5. Did not look into how to access to funding opportunities

6. Other (specify)…….

33. Do you consider yourself to have enough opportunities to participate in conferences and workshops?
    1. Yes
    2. No

34. If yes, mention which ones?
    1. Local conferences and workshops
    2. International conferences and workshops
    3. Both local and international conferences and workshops
    4. Not applicable (***select this option if the response to question 30 is "No***")

35. If the answer is no, mention why?
    1. Did not receive any funding
    2. Did not receive any information on opportunities
    3. Could not find information on opportunity
    4. Was not authorized
    5. Did not look into how to access such opportunities
    6. Other, specify…….

36. How frequently do you engage in presentation to the public at a scientific conference
    1. Never
    2. Once per year
    3. Twice per year
    4. Four times per year
    5. More than four times per year

37. Have you ever had a period of time during your professional training or in your career where it was difficult for you to fulfill your professional responsibilities because of your personal responsibilities?
    1. Yes
    2. No

38. If yes, what were the main reasons?
    1. Care for children
    2. Care for family member (other than children)
    3. Illness/accident
    4. Burn out
    5. Depression
    6. Change in a marital situation (for example divorce)
    7. Death in family or of closely related person
    8. Other, specify……………
    9. Not applicable (**select this option if your answer to question 42 is "No**")

39. Have you ever received any awards for your accomplishments?
    1. Yes
    2. NO

40. Have you ever been invited to participate in committees by the Ministry responsible for Science, Technology and Innovation (STI) in Zambia
    1. Yes
    2. No

41. Have you applied for a research grants before?
    1. Yes
    2. No

42. If yes, how many times have you applied for grants from national and international institutions over the past 2 years? Indicate number:……………..

43. If yes, what was your role?
    1. Principal investigator
    2. Part of a team

44. Where did you apply?
    1. National funding institution
    2. International funding institution

3. Both national and international funding institution

45. How many national grants have you won? Indicate number and amount……………………...

46. How many international grants have you won? Indicate number and amount…………………

Gender equality in research

47. In your opinion, can female researchers be as successful in winning research grants as their male folks?
    1. Yes
    2. No
    3. I don't know

48. In your opinion, can female researchers be as successful in publishing their scientific work as their male folks?
    1. Yes
    2. No
    3. I don't know

49. Do you think the field of science and research is equally suited for both men and women?
    1. More suitable for men
    2. More suitable for women
    3. Suitable for both men and women

50. Are you aware of instances of a pay gap between men and women who have the same responsibilities in your organization?
    1. Yes
    2. No

Harassment at a place of work

Harassment can take any form such as verbal, physical, psychological, sexual or other forms of abuse by a colleague, superior of the same or opposite sex. It can also include being denied access to certain services or privileges on account of sex, place of origin, ethnicity race and other stereotypes

51. Have you had any experience of harassment at your current or a former workplace?
    1. Yes I have witnessed it
    2. Yes I have heard second hand reports
    3. No
    4. I can't tell

52. If yes, what form of harassment did you experience?
    1. Verbal abuse from a colleague
    2. Verbal abuse by a superior
    3. Denied access to research funds
    4. Sexual abuse
    5. Others, Specify…………

53. Have you ever left a job on account of harassment?
    1. Yes
    2. No
54. If yes, to what extent did harassment at work contribute to your leaving the job?
    1. It was a contributing factor
    2. It was not a factor
    3. Not applicable
    4. I have not left

THE END
