# Supplementary material for: Gender integration and female participation in scientific and health research in Zambia: a descriptive cross-sectional study protocol
Source: BMJ Open. 2023 Mar 6;13(3):e064139. doi: 10.1136/bmjopen-2022-064139 (PMC9990657; doi:10.1136/bmjopen-2022-064139)
Supplement: Supplementary data [file bmjopen-2022-064139supp003.pdf]

INTERVIEW GUIDE FOR ACADEMIC INSTITUTIONS

Gender dimensions and factors affecting female participation in Science and Health Research in Zambia

Theme 1: Policies on Integration of Gender Dimension in scientific and health research

Sub-theme 1.1: Policies on student recruitment

Probe for:

- Available policies on student recruitment in scientific disciplines at undergraduate level
- Available policies on student recruitment in scientific disciplines at postgraduate level
- Dissemination of available policies in the institution
- Extent to which available policies are implemented in the institution
- Policy implementation with regard to promotion of academic staff

Sub-theme 1.2: Policies on staff recruitment and promotion

Probe for:

- Available policies on staff recruitment and promotion in the institution
- Integration of gender in the recruitment and promotion of staff to academic positions
- Integration of gender in the recruitment and promotion of staff to administrative position
- Dissemination of available policies in the institution
- Extent to which available policies are implemented in the institution

Sub-theme 1.3: Policies on Integration of Gender Dimension in Scientific and health research

Probe for:

- Available policies on scientific research and innovation in the institution (eg policy on student recruitment at undergraduate, postgraduate, mention available policies)
- Integration of gender in the available policies
- Mention specific aspects of gender integration in the policies
- Extent to which the available policies are disseminated in the institution
- Extent to which the available policies are implemented in the institution

Theme 2: Participation in Scientific and Health Research

Probe for:

- Extent to which staff in the institution are involved in Scientific and research n
- Extent to which gender dimension is considered in staff participation in scientific and research
- (opportunities for research grants; award of internal and external grants, attending local and international conferences)
- Extent to which gender dimension is considered in staff career path in science and research
- Extent to which gender dimension is considered in staff promotion in scientific and health research
- Suggestions for improving gender integration and female participation in scientific and health research

Theme 3: Factors influencing gender integration and female participation in scientific and health research

Probe for both facilitators and barriers:

- Institutional norms and prejudices on female participation and gender integration in Scientific and health research
- Decision-making on award of research grants in the institution
- Attitude towards female participation in scientific and health research
- Structural factors in the institution (eg, workload, hierarchy)
- Family and personal factors (marriage, religious)

INTERVIEW GUIDE FOR RESAERCH INSTITUTIONS

Exploring the gender dimensions and factors affecting female participation in scientific and health research in Zambia

Theme 1: Policies on Integration of Gender Dimension in scientific and health research

Sub-theme 1.1: Policies on staff recruitment and promotion

Probe for:

- Available policies on staff recruitment and promotion in the institution
- Integration of gender in the recruitment and promotion of staff to scientific and
- Integration of gender in the recruitment and promotion of staff to administrative position
- Dissemination of available policies in the institution
- Extent to which available policies are implemented in the institution

Sub-theme 1.2: Policies on Integration of Gender Dimension in Scientific and health research

Probe for:

- Available policies on scientific research and innovation in the institution (eg policy on recruitment mention available policies)
- Integration of gender in the available policies
- Mention specific aspects of gender integration in the policies
- Extent to which the available policies are disseminated in the institution
- Extent to which the available policies are implemented in the institution

Theme 2: Participation in Scientific and Research

Probe for:

- Extent to which staff in the institution are involved in Scientific research and Innovation
- Extent to which gender dimension is considered in scientific research and Innovation and grant opportunities (award of internal and external grants, attending local and international conferences)
- Extent to which gender dimension is considered in staff career path in Scientific research and Innovation (training opportunities, etc)

- Extent to which gender dimension is considered in staff promotion
- Suggestions for improving gender integration and female participation in the institutions

Theme 3: Factors influencing gender integration and female participation in scientific and health research

Probe for both facilitators and barriers:

- Institutional norms and prejudices on female participation and gender integration in Scientific and health research
- Decision-making on award of research grants in the institution
- Attitude towards female participation in STI in academia and research
- Structural factors in the institution (eg, workload, hierarchy)
- Family and personal factors (marriage, religious)
