# Supplementary material for: Gender integration and female participation in scientific and health research in Zambia: a descriptive cross-sectional study protocol
Source: BMJ Open. 2023 Mar 6;13(3):e064139. doi: 10.1136/bmjopen-2022-064139 (PMC9990657; doi:10.1136/bmjopen-2022-064139)
Supplement: Supplementary data [file bmjopen-2022-064139supp004.pdf]

A. **Research Grants: Local Funding (source within Zambia)**

Name of the School:…………………………………………

**Year**	**2017**		**2018**		**2019**		**2020**		**2021**		**Total**		**Grand Total**
	**M**	**F**	**M**	**F**	**M**	**F**	**M**	**F**	**M**	**F**	**M**	**F**	
SDF													
Lecturer 3													
Lecturer 2													
Lecturer 1													
Associate Professor													
Full Professor													
Total for the year													

B. **Research Grants: International Funding (Foreign Source)**

Name of the School:…………………………………………

**Year**	**2017**		**2018**		**2019**		**2020**		**2021**		**Total**		**Grand Total**
	**M**	**F**	**M**	**F**	**M**	**F**	**M**	**F**	**M**	**F**	**M**	**F**	
SDF													
Lecturer 3													
Lecturer 2													
Lecturer 1													
Associate Professor													
Full Professor													
Total for the year													

C. **Publications in international Peer Reviewed Journals**

Name of the School:…………………………………………

**Year**	**2017**		**2018**		**2019**		**2020**		**2021**		**Total**		**Grand Total**
	**M**	**F**	**M**	**F**	**M**	**F**	**M**	**F**	**M**	**F**	**M**	**F**	
SDF													
Lecturer 3													
Lecturer 2													
Lecturer 1													
Associate Professor													
Full Professor													
Total for the year													
