# Supplementary material for: Gender integration and female participation in scientific and health research in Zambia: a descriptive cross-sectional study protocol
Source: BMJ Open. 2023 Mar 6;13(3):e064139. doi: 10.1136/bmjopen-2022-064139 (PMC9990657; doi:10.1136/bmjopen-2022-064139)
Supplement: Supplementary data [file bmjopen-2022-064139supp005.pdf]

Table 2: Summary of data collection techniques

Objective	Technique	Target institution/ group	Instrument
Explore gender integration in science and research	Desk and literature review	UNZA<br>Copperbelt University (CBU)<br>Ministry of Higher Education<br>Ministry of Gender	Checklist
Perspective on factors affecting female participation in science and research	IDI	UNZA<br>CBU<br>Mount Makulu<br>TDRC	Interview guide
Identify the factors that affect female participation in science and research	Survey	UNZA<br>CBU	Questionnaire
