# Supplementary material for: Gender integration and female participation in scientific and health research in Zambia: a descriptive cross-sectional study protocol
Source: BMJ Open. 2023 Mar 6;13(3):e064139. doi: 10.1136/bmjopen-2022-064139 (PMC9990657; doi:10.1136/bmjopen-2022-064139)
Supplement: Supplementary data [file bmjopen-2022-064139supp006.pdf]

Informed Consent Form

Exploring the gender dimensions and factors affecting female participation in Science, Technology and Innovation (STI) in Academia, Industry-Research & Development in Zambia

Background

Hello, my name is __________________________ I am working with the Levy Mwanawasa Medical University and University of Zambia. I am part of the research team on "Exploring the gender dimensions and factors affecting female participation in Science, Technology and Innovation (STI) in Academia, Industry-Research & Development in Zambia". Despite the Zambian government making remarkable progress on prioritizing gender mainstreaming, female participation in science, innovation, research and development is still low. Moreover, information on the integration of gender dimension and the factors affecting inclusion of men and women in Science, innovation, academia, research & development is scant. To this end, the National Science Technology Council has funded this study.

Purpose: The aim of this research project is to explore the integration of gender dimensions and the factors that influence female participation in STI in academia-industry-research & development in the country in order to bridge the knowledge gaps and inform policy and programming to support the advancement of gender equality in STI in the country. We would like to speak with you because you are …………….... We would like to understand your opinion and perspectives on the extent to which females are involved in and the factors influencing female participation in science-related fields of study in Zambia.

Process: You will be one of approximately 450 respondents asked to participate in this discussion. We would like to ask you some questions in order to understand your opinions and perspectives on this subject. The discussion should take no longer than 60 minutes. To ensure that I don't miss or forget anything during the discussion, your responses to the various questions will be recorded using a digital recorder. We will not write your name, instead we will ask you to sign on this form; we will keep your signature private. Responses from the various questions will be summarised, analysed and a report written. When we write reports from this discussion, we will not show what you said nor are we going to use your name or signature at any time.

Potential Benefits: You will receive no direct benefit from your participation in this discussion. However, your participation may help the Zambian government to understand the integration of gender dimensions and the factors that influence female participation in STI in academia-industry-research & development in the country. This information will help to bridge the knowledge gaps and in turn, inform policy and programming to support the advancement of gender equality in STI in the country.

Risks and Discomforts: The risks of taking part in this study are that other people will hear your responses. It is important that you do not share anything that you are not comfortable with. If you or someone in your family had a bad experience, it may be difficult or uncomfortable to remember or share it. You do not have to respond to any question unless you feel comfortable doing so. You are free to stop the discussion at any time if you need to.
.

Alternatives: You can choose not to take part in this discussion. If you decide not to take part or withdraw from this discussion, you will not suffer any penalty or lose any benefits to which you are entitled.

Participant Costs and Payments: You will not be paid to participate in this discussion. There are no costs to you for participating.

Participant's Rights: By agreeing to participate in this discussion, you do not waive any of your legal rights. Giving consent means that you have heard or read the information about this discussion and that you agree to participate. You will be given a copy of this form to keep. If you have questions or concerns at any time, you can contact the Principal Investigator, Dr. Sialubanje Cephas on +260-977-441 273, the Co-Investigator, Prof Joseph Mumba Zulu on +260 971 591 388 or any of the staff from the University of Zambia Biomedical Research Ethics Committee on +260-21-1-256067.

Right to Refuse or Withdraw: Taking part in this discussion is voluntary. You have the right to refuse to take part. If you decide

to be in this discussion, and then change your mind, you can withdraw from it at any time and to skip questions you may deem personal or otherwise without any repercussions. Your participation is voluntary. If you choose to take part, you have the right to stop the discussion at any time.

Confidentiality: The results of this study will be kept strictly confidential, and used only for research purposes. Your identity will be concealed in as far as the law allows. Your name will not appear anywhere on the coded forms with the information. Paper and computer records will be kept under lock and key and with password protection respectively. The interviewer has discussed this information with me and offered to answer my questions. For any further questions, I may contact the Chairperson, UNZABREC on the following details___________________

STATEMENT OF CONSENT/ASSENT

.... has described to me what is going to be done, the risks, the benefits involved and my rights regarding this study. I understand that my decision to participate in this study will not alter my usual medical care. In the use of this information, my identity will be concealed. I am aware that I may withdraw at anytime. I understand that by signing this form, I do not waive any of my legal rights but merely indicate that I have been informed about the research study in which I am voluntarily agreeing to participate. A copy of this form will be provided to me.

Name:………………………….Signature of participant ……………………..Age..……………
Date (DD/MM/YY)……………………………

Name of Witness……………………………….. Signature of Witness…………………………
Date (DD/MM/YY)…………………………………………….

Name…………………………Signature of parent or guardian for minors ……………………
Date(DD/MM/YY)…………………………

Name……………………………………..Signature of Interviewer …………………………Date (DD/MM/YY)………………………...

If you have any further questions please contact the University of Zambia Biomedical Research Ethics Committee

Telephone: 256067

Telegrams: UNZA, LUSAKA

Telex: UNZALU ZA 44370

Fax: + 260-1-250753

E-mail: unzarec@unza.zm

Ridgeway Campus

P.O. Box 50110

Lusaka, Zambia

Assurance No. FWA00000338 **IRB00001131 of IOR G0000774**

By signing below you are agreeing to participate in the discussion which indicates that you have read this consent form (or have had it read to you), that your questions have been answered to your satisfaction, and that you voluntarily agree to participate in this research study. You may keep a copy of this for your records.

Signature or thumb print: ____

Date: ____

Signature of Impartial Witness: ____

Date: ____

2
