# Supplementary material for: Gender integration and female participation in scientific and health research in Zambia: a descriptive cross-sectional study protocol
Source: BMJ Open. 2023 Mar 6;13(3):e064139. doi: 10.1136/bmjopen-2022-064139 (PMC9990657; doi:10.1136/bmjopen-2022-064139)
Supplement: Author's manuscript [file bmjopen-2022-064139.draft_revisions.pdf]

BMJ Open is committed to open peer review. As part of this commitment we make the peer review history of every article we publish publicly available.

When an article is published we post the peer reviewers' comments and the authors' responses online. We also post the versions of the paper that were used during peer review. These are the versions that the peer review comments apply to.

The versions of the paper that follow are the versions that were submitted during the peer review process. They are not the versions of record or the final published versions. They should not be cited or distributed as the published version of this manuscript.

BMJ Open is an open access journal and the full, final, typeset and author-corrected version of record of the manuscript is available on our site with no access controls, subscription charges or pay-per-view fees (http://bmjopen.bmj.com).

If you have any questions on BMJ Open's open peer review process please email info.bmjopen@bmj.com

BMJ Open

Gender integration and female participation in scientific and health research in Zambia: A descriptive mixed methods cross-sectional study

Journal:	*BMJ Open*
Manuscript ID	bmjopen-2022-064139
Article Type:	Protocol
Date Submitted by the Author:	24-Apr-2022
Complete List of Authors:	Sialubanje, Cephas; Levy Mwanawasa Medical University, Levy Mwanawasa Medical University<br>Sumbwa, Phyllis; University of Zambia<br>Zulu, Nyondwa; University of Zambia, School of Education<br>Mwanza, Nchimunya; University of Zambia, School of Public Health<br>Chavula, malizgani; University of Zambia, School of Public Health<br>Zulu, Joseph; University of Zambia, School of Public Health
Keywords:	HEALTH SERVICES ADMINISTRATION & MANAGEMENT, Health policy < HEALTH SERVICES ADMINISTRATION & MANAGEMENT, Human resource management < HEALTH SERVICES ADMINISTRATION & MANAGEMENT

SCHOLARONE™
Manuscripts

Gender integration and female participation in scientific and health research in Zambia: A descriptive mixed methods cross-sectional study

Cephas Sialubanje[1], Phyllis Ingutu Sumbwa[2], Nyondwa Zulu[2], Nchimunya B. Mwanza[3], Malizgani Paul Chavula[3], Joseph M. Zulu[3]

[1]School of Public Health, Levy Mwanawasa Medical University, P.O. Box 33991, Lusaka, Zambia

[2] School of Education, University of Zambia, P.O. Box 323791, Lusaka, Zambia

[3]School of Public Health, University of Zambia, P.O. Box 32379, Lusaka, Zambia

For peer review only

Abstract

Introduction: Despite the Zambian government making progress on prioritizing gender mainstreaming, female participation in science, technology and innovation (STI) in academia, research and development is still low. This study aims to determine the integration of gender dimensions and the factors that influence female participation in scientific and health research in Zambia.

Methods and analysis: We propose a descriptive mixed methods cross-sectional study design employing record review, in-depth interviews (IDIs) and survey as data collection techniques. Twenty schools offering science-based programs will be purposively selected from the university of Zambia and Copperbelt University. Two research institutions in Zambia, Tropical disease research centre and Mount Makulu Agricultural Research station will also be included. Survey respondents will include a random sample of 423 academic and research staff from the selected schools. In addition, a total of 30 IDIs will be conducted with staff and heads of selected schools and research institutions. Data collection will be conducted over a 12 months period. In-depth literature search and record review on gender dimensions in science and health research will be conducted to gain insight into the subject matter and inform research instrument design. Survey data and IDIs will be collected using a structured paper-based questionnaire and semi structured interview guide, respectively. Descriptive statistics will be computed to summarise respondents' characteristics. Bivariate analysis (chi square and independent t-test) and multivariate regression analysis will be conducted to test the association and identify factors influencing female participation in science and health research (adjusted odds ratios, $p<0.05$). An inductive approach will be used to analyse qualitative data using N-vivo. Survey and IDI will be corroborated.

Ethics and dissemination: Ethical approval was obtained from the University of Zambia Biomedical Research Ethics Committee. Study findings will be disseminated through a written report, stakeholder meetings and publication in a peer reviewed international journal.

Word count: 298

Key words: gender mainstreaming, Science, Health research sustainable development goals

For peer review only

Strengths and limitations of the study

- Use of mixed methods cross-sectional design employing in-depth literature review, survey and in-depth interview techniques increases internal validity of the study
- Use of probability proportional to size (PPS) and random sampling techniques to select schools and study participants, respectively, minimizes bias due to selection bias
- Training of data collectors minimizes measurement error
- Cross-sectional design of the study does not allow for causal inference
- Selection of all the 16 schools offering science-based programs from only two public institutions in may introduce selection bias.

For peer review only

1. Introduction

1.1. Background

Gender mainstreaming is a public policy concept of assessing different implications of any public policy action, legislation and programs on people of different genders [1]. The concept was first proposed at the 1985 third World Conference on Women in Nairobi, Kenya [1]. It has been adopted into the United Nations' (UN's) development community as a globally accepted strategy for promoting gender equality [2] and ensuring that all UN member states achieve the 50 percent gender parity−through eempowering all women and girls, ending all forms of discrimination against women and girls everywhere and ensuring women's full and effective participation and equal opportunities at all levels of decision making [3]. Gender equality ensures that women have the same opportunities in life as men, including the ability to participate in the public sphere [9]. It also ensures that gender perspectives and goals are central to all activities including policy development, research, advocacy/ dialogue, legislation, resource allocation, planning, implementation and monitoring of programmes and projects [3]. Gender equality also ensures equitable distribution of benefits from the development process for boys, girls, men and women. It is at the heart of strategies and interventions driving the active and meaningful participation of both women and men in the development agenda, and ensures consistent empowerment of women and girls. It is a basic requirement for measuring progress on SDGs and constitutes a key aspect within the principle of leaving no one behind, without which the SDGs cannot be achieved [4]. Both gender equality and women's empowerment are integral to realizing the 2030 Agenda for sustainable development [3-5] and all the UN sustainable development goals (SDGs) [3-5] which were agreed upon in the UN sustainable development groups (UNSDG) in the UN Country Team (UNCT) System-wide Action Plan (SWAP).

Zambia is one of the UN member states that have adopted the gender mainstreaming concept and developed a gender national gender policy [6]. The Zambian national gender policy [6] focuses on attainment of gender equality in the development process and providing for equal opportunities for women and men to actively participate and contribute to their fullest ability. The policy also focuses on equitable benefit from national development.

Although the Zambian government has made significant progress in adopting the policy [7] and increasing female enrolment in secondary and higher education as well as their representation in decision-making positions [8-10]_female representation and participation in Science, technology and innovation (STI) in academia, research and development is still low and far from achieving the UN 50 percent gender parity

For peer review only

target [11]. Reports and studies conducted in Zambia [8,9] reveal unequal gender representation in STI in academia-industry-research & development, with women continuing to lag behind their male counterparts, and their access and retention in tertiary education remains a challenge. For example, the Ministry of Gender and Child Development report shows that in 2011 [10], females only comprised 39.1% of the combined total enrollment at the four Zambian Public Universities (University of Zambia, Copperbelt, Mulungushi and Nkrumah). A European Commission (EU) for Research and Innovation (2016) report [11] shows that the proportion of women enrolling for graduate studies in Science, Technology, Engineering and Mathematics (STEM) programmes is low at less than 20% [11], and women comprise less than 25% of the doctoral graduates in the field of engineering and computer Science [12]. Similarly, the UNESCO reports [13,14] shows that the representation of women in research and development at the global level is low at 30%.

Reasons for the low female participation in this field are not well understood. Most published studies on the subject relate to developed countries such as the United States and Europe [15]. The few published studies are on information, communication and technology (ICT) [16-20] in secondary in and higher institutions of learning, which are not applicable to STI in academia, research and development. Thus, the actual gender context with regard to integration of gender dynamics and the issues affecting female participation in STI in academia, research and development in the country are not well understood despite the growing recognition of the importance of gender equality in the country. A study is therefore required to answer the following questions in order to bridge the knowledge gaps on this subject:

1) To what extent is gender mainstreaming integrated in science and health research?
2) What is the proportion of females participating in science and health research in Zambia?
3) What factors influence female participation in science and health research in the country?

1.2. Objectives

This study aims to explore the integration of gender dimensions and the factors that affect female participation in scientific and health research the country. The specific objectives are:

1) Explore gender integration in scientific and health research
2) Explore female participation in scientific and health research
3) Identify the factors that affect female participation in scientific and health research

For peer review only

This knowledge is important for informing policy and programming for the advancement of gender equality, equal opportunities and participation in science, health research and development in order to promote quality scientific and health outcomes and technological excellence. Incorporating gender dimensions and deliberately encouraging females to participate in science and health research is crucial in realizing the key role that females can play in the development and advancement of science, and their commitment to inclusive science and research development. Gender equality in science and research is not only a matter of fairness but important for enhancing the recruitment of the most talented individuals, irrespective of gender, and for tapping a partially unexploited resource [21, 22]. It is a way to promote scientific and technological excellence rather than just improving opportunities for women [23, 24]. A more inclusive workforce is likely to be more innovative and productive than one which is less so [25]. Moreover, having scientists and researchers with diverse backgrounds, interests, and cultures assures better scientific research and the best use of those results [23-26]. Mainstreaming gender in science and health research is crucially important for various reasons. First, women are the main driving labor force in most African countries, making a vital contribution to the economy. Empirical evidence shows that if women had equal access to productive resources such as science, technology, research, or financial services, the gender productivity gap would almost disappear and African countries, including Zambia, could significantly increase their production by just closing the gender gap across economic, academic and scientific divide. Second, increasing women's participation in STI has the potential to lead to higher GDP growth through increases in higher-skilled women in the labor force and women's employment in high-productivity sectors. Gender equality and empowerment of females to participate in science and health research can also boost women's role as leaders in the field. [27.28].

2. Methods

2.1. Study design

We propose to conduct a descriptive mixed methods cross-sectional study design employing record review, in-depth interviews (IDIs) and survey as data collection techniques. Use of qualitative and quantitative data collection and analysis techniques will make the study comprehensive and allow for triangulation and corroboration of the findings. This will in turn increase the internal validity of the findings [29,30]

2.2. Setting

The study will be conducted at the two public institutions of higher learning in Zambia (university of Zambia, Copperbelt and Mulungushi universities). A total of sixteen schools (ten from the university of Zambia and six from Copperbelt university) offering science and health-related programs will be purposively selected from each institution. The University of Zambia (UNZA) is located in Lusaka, the capital city of Zambia. It is the

For peer review only - http://bmjopen.bmj.com/site/about/guidelines.xhtml

largest and oldest public university in the country. It was founded in 1965 and established by Act of Parliament No. 66 of 1965. The first intake of students took place on 17th March 1966. It offers a number of academic programs at Diploma, Undergraduate and postgraduate levels. The university has 13 faculties including Agricultural Sciences, Education, Engineering, Graduate School of Business, Health Sciences, Law, Medicine, Mining, Public Health, Natural Sciences, Nursing Sciences, Social Sciences and Humanities, Veterinary Medicine. It also has the directorate of research and graduate studies. The Copperbelt University is the second largest and oldest public university. It is located in Kitwe, Zambia. It has 12 schools including: school of the built environment, school of Business, Dag Hammarskjold Institute for peace and conflict resolution, Directorate of distance education and open learning, School of Engineering, School of Graduate studies, School of Mathematics and Natural Science, School of Medicine, School of Mines and Mineral Resources, School of Natural Resources, School of Information and Communication Technology, and School of Humanities.

A total of ten school offering science- and health-related programs will be purposively selected from UNZA, namely: Medicine, Public Health, Nursing Sciences, Health Sciences, Veterinary Medicine, Mines, Engineering, Natural Sciences, Agricultural Sciences, Directorate of research and Postgraduate studies. All the six schools offering science and health-related programs at the Copperbelt university will be included in the study: School of Engineering, School of Mathematics and Natural Science, School of Medicine, School of Mines and Mineral Resources, School of Natural Resources, and School of Graduate studies. In addition, two research institutions Tropical disease research centre (TDRC) from Ndola Zambia, and Mount Makulu Agricultural Research station in Chilanga were included in the study.

2.3. Participants and sampling process

Survey participants will comprise a randomly selected sample of 423 male and female academic and research staff from the 16 selected schools in the two academic institutions (223 from UNZA and 200 from the Copperbelt university). To be eligible, survey participants will be:

- Lecturers and researchers at any academic rank (lecturer three to full professor)
- Having worked in the selected institution for at least 3 months

IDI participants will include staff from the selected schools and the two research institutions. To be eligible, IDI participants will be:

- Registrar or Assistant Registra

- School Dean or Assistant Dean
- Head of Department or Section Head
- Director of directorate of research and graduate studies (DRGS)
- Head or assistant head of selected research institution
- Section head from selected research institution

Survey participants will be selected using a stratified sampling technique. First, the number of staff from each school will be obtained. To ensure that all schools are proportionately represented, probability proportional to size (PPS) will be conducted to determine how many participants will be selected from each school. Next, a list of all the lecturers in each of the selected schools from will be compiled to serve as a sampling frame.. To ensure that both male and female lecturers have an equal chance of being selected, separate lists will be prepared for each sex. Using a random number generator, random sampling will be done and study participants identified. The process will be done for each school at the two institutions.

Purposive sampling will be used to recruit IDI participants from the selected institutions. In order to compare and contrast the respondents' views, both male and female participants will be included. We plan to conduct a total of thirty IDIs. However, the actual number of IDIs to be conducted will be determined by the point of saturation [29, 30]. In order to get variability in responses, compare and contrast the similarities and differences in participant perspectives on the subject, efforts will be made to ensure that both male and female respondents are included in the IDIs and questionnaire survey. Efforts will also be made to have a mix of survey participants from the lowest (lecturer 3) to the highest rank (Full professor).

2.4. Sample size estimation

The sample size for the survey respondents will be calculated using *a single population proportion formula* as shown below [31]. The single population proportion formula is used to esmiate the sample size for a one group of respondnets, when no comparison group is required [31]. Since the proportion of females having the characteristic of interest (that is, the proportion of females participating in STI) is not known, an assumption of 50% of the study population having the characteristic of interest will be made [31]. With this assumption, the sample size will be calculated using the formula below:

$$n=[Z^2 * (P(1-P)]/ d^2*DEFF$$

Where, n=sample size;

For peer review only - http://bmjopen.bmj.com/site/about/guidelines.xhtml

Z=Z-score at 95% confidence level (1.96), and alpha level of 5%

P=Proportion of population with a characteristic of interest (in this case female partication in STI) d=margin of error at 5%.

DEFF= Design Effect, given that the study sites are relatively near to each other, there would be some interaction and some intra cluster correlation, thus the design effect is equal to 1.

=1.96*1.96 [0.5X0.5]/(0.05*0.05)

=384.16.

Allowing for a 10% nonresponse rate (NRR), a total sample size of **423 survey** will be required to measure the proportion of females participating in STI and estimate the determinants of female participation in STI with 95% confidence. Thus, **n=423**

2.5. Variables

The study variables will include the outcome and independent variables as follows:

Outcome variable: Female participation in science and health research and development

Independent variables:

a) Demographic information: age, sex, number of children
b) Socioeconomic information: level of education, field of study

2.6. Data sources and collection techniques

In order to explore gender integration in scientific and health research, we propose to conduct an in-depth search and review of the literature including: a) enrolment and graduation data from the two selected institutions, b) administrative data on staffing profile in the schools; c) data on both external and internal research grants in the institutions, d) data on local grants from Science Granting Councils, e) review of national and institutional documents on gender policy and integration from the two institutions and relevant institutions, Gender and Higher Education and Un agencies (UN women and UNESCO women in Science) and the two research institutions (Mount Makulu Agricultural Research Station in Chilanga, TDRC in Ndola).

To determine the factors influencing gender integration and female participation in scientific and health research, a survey using a structured questionnaires will be conducted among a randomly selected sample

For peer review only

of 423 lecturers from the selected 16 schools. To explore the respondents' perspectives on gender integration and female participation in science and research, IDIs will be conducted with registrars, heads of schools, head of department, head of DRGS from the two institutions. Other participants will include head of selected research institutions as shown in table 1 (**Supplimentary material 1**).

2.7. Data collection procedures

Data collection will be conducted over a 12 months period. IDis will be conducted by a pair of trained reseaerch assistants with the supervision of the research team members. Survey questionnaires will be administered by the Master of Public (MPH) students from the School of Public Health, University of Zambia. Training of research assistants will be conducted by the principal investigator (PIs) and the co-investigator. Training will take a total of five days, and will be in two phases: theory and field work. Phase one will include theory in research and will take 3 days. The topics will include: 1) basic principles of qualitative and quantitative research, 2) the purpose and objectives of the study, 3) data collection and interviewing techniques, 4) research ethics and 5) informed consent in human subjects' research. Phase two will be field practical and will include pre-testing the data collection tools and consent form, and will take 2 days, after which the instruments will be revised based on the collected and analysed data and feedback from the research assistants. Each IDI and self administered questionnaire is expected to last between 30 and 45 min. To allow for triangulation and validation of findings, both qualitative and quantitative studies will be conducted simultaneously.

2.8. Data collection instruments

A paper based standard, structured and pre-tested questionnaire (**Supplimentary material 2**) will be prepared and used to collect survey data. IDIs will be collected using an interview guide (**Supplimentary material 3**). A checklist (**supplimentary material 4**) will be prepared for record review. The questionnaire, interview guide and checklist will be in English and developed by the research team. The same iinterview guide will be prepared for lecturers and managers from the research institutions. The interview guide will be developed based on specific objectives: a) perspectives on integration of gender in science and research, b) participation/involvement in science and research, c) factors influencing female participation in science and research. The survey questionnaire will also have the same sections but will include an additional section on the sociodemographic and economic characteristics. Before each IDI, respondent sociodemographic data including, age, sex, marital status, occupation, level of education and level of monthly income, will be

For peer review only

collected using a short questionnaire. Interviews will be recorded on digital audio-recorders and notes will be taken by research assistants. The data collection tools are summarised in table 2 (**Supplimentary material 5**).

2.9. Bias

To minimize bias and ensure quality of data collection process, the following measures will be taken: a) the sample size estimation has been determined using scientific methods to minimize random error; b) to minimize measurement error, data collection instruments will be pre-tested and revised accordingly, c) sampling procedures will be followed to minimize selection bias; d) to minimize measurement error, research assistants will be trained; e) survey data will be entered and analysed by an independent and competent person to reduce data entry errors; e) coding and analysis of qualitative data will be done by two research team members to increase internal validity. During fieldwork, the team will ensure quality of both quantitative and qualitative data collection process by a) an accompanying note will be attached to each questionnaire to ensure that questionnaires are completely filled in, a) taking extensive notes during key informant interviews, b) comparing notes each day after the interviews.

2.10. Statistical methods and data analysis

Quantitative/survey data will be analysed using SPSS version 21 (IBM, Armonk, NY, USA). Descriptive statistics and frequencies will be used to summarise survey data. Categorical data will be presented using percentages, quantittaive data will be presented using the mean and standard deviation (or median and interquartile range). Reliability test for internal consistency using Cronbach's alpha will be conducted on each of the items. Items that show strong internal consistency (Cronbach's alpha 0.60 or $r > 0.40$) will be combined and averaged into one variable based on factor analysis. Bivariate analysis will be done using Pearson's correlation to test the association between the independent and outcome variables. Multivariate regression analysis will be conducted to identify predictors of female participation in science and research ($r>0.10$, $p<0.05$).

Qualitative data analysis will be done using an inductive approach which ensures that sub-themes are derived from the pre-determined themes by content-analysis and grouping all similar statements made with respect to particular themes. The voice recordings from the key informant interviews will be transcribed by the research assistants. After verification of accuracy in transcription, each transcript will then be thoroughly read by one research assistant while the other one will be listening to the corresponding voice recording. Each

transcript will then be compared with the hand-written field notes that the research assistants will prepare during the IDIs. After proof-reading and making corrections, the transcripts will be saved on a password-protected file computer kept by the PI for safety. The Word documents will then be imported into Nvivo 11 MAC for coding and analysis. The exported data will then be coded using a codebook and guiding framework, and the categories and key sub-themes will be identified.

2.11. Patient and public involvement

The study design was determined by the call for application for a research grant on gender dimensions in Science, Technology and innovation under the strategic research fund. Thus, the participants and the public were not directly involved in the conceptualisation and design of the study. Nevertheless, selection of the 16 schools from the two institutions was done in consultation with the authorities from the Ministries of Higher Education and Gender. Further, recruitment of study participants will be done in collaboration with the respective School Deans and their assistants. To inform the institutions about the study, a letter will be written to the registrars and heads of the research institutions. Next, meetings will be held with the two respective School Deans and the heads of the two research institutions to discuss the study and recruitment of survey and IDI participants. Finally, a report will be written and shared with key stakeholders, including the funding organisation and Ministry of Higher Education and Gender. In addition, a dissemination meeting will be held in Lusaka to share the results with the two Ministries and heads of the participating schools and institutions.

2.12. Ethical considerations

Ethical clearance and approval was obtained from the University of Zambia Biomedical Research Ethics Committee (UNZA BREC 1674-2021; **supplementary material 5**). After ethical clearance and permission to conduct the study, respondents will be recruited with help from the study site managers. Potential participants will be contacted on phone or by email. The purpose, process and duration of the study, benefits and associated risks, privacy and confidentiality will be explained. Those who accept to participate in the study will be enlisted in the study and asked to sign the consent form (**Patient Consent Form 1**) that will be attached to the questionnaire. The document will be shared by the research team and respondents will be asked to sign the consent form.

Written informed consent will be obtained from each IDI participant. Those who cannot read or write will be asked to mark with an 'X'. Participants below 18 years old will require both parental consent and assent from parents.

For peer review only

Potential risks and mitigation

This study poses minimal risk to study participants. Research assistants will not administer the questionnaire; questionnaires will be delivered to the respondents' office for self-administration. Completed questionnaires will be sealed in an envelope for research assistants to collect. Participation in the study may cause some discomfort from answering certain questions, particularly if the respondent had a negative gender-related experience. To reduce the risk of disclosure of personal or sensitive information, data collectors will be trained to ensure that they advise participants not to share anything that they are not comfortable with; and that they do not have to respond to any question unless they feel comfortable doing so. Since the questionnaire will be self-administered, respondents will have minimal risk of COVID-19 infection.

The study may expose IDI participants and study team members to COVID-19 infection. Bio-safety measures will be taken during data collection including consistent wearing face masks, social distancing and conducting virtual interviews to minimize the risk of infection to the research team and study participants. In addition, data collectors and study participants will be encouraged to consistently use face masks and hand sanitisers before and after each interview. Used face masks will be disposed of according to the health guidelines. .Data collectors are also trained to minimise any potential discomfort or harm to all participants during all study activities to the greatest extent possible. The study team will reduce any waiting by participants by scheduling appointments during times convenient to participants and interviews are kept to as short a time as possible. To ensure data quality, weekly research meetings will be held to ensure effective and efficient execution of the project.

2.13. Potential benefits

There are no direct individual benefits to participating in the study. If effective, the proposed study findings will be disseminated among the stakeholders and likely to lead to design of evidence informed strategies to improve female participation in science and research.

2.14. Participant confidentiality

Confidentiality of collected data from study participants will be assured throughout the study. The interview will be carried out in participant's office or place of choice. Data collection will not be conducted until we can confirm that the time and location are acceptable to the participants. On completion of IDI and survey, all survey data will be entered in excel and stored on a secure pass-code protected computer kept by the PI. IDI audio recordings will be transcribed and stored on the same computer. Both survey and IDI data will be de-

For peer review only

identified and the linking file with identifiable data and basic demographics will be stored in a separate file. Only the PI and some identified research teams will have access to the identifiable data. All analyses will be conducted on de-identified data.

2.15. Limitations

This study has several limitations. First, the study will only include 423 survey respondents and 30 IDI participants from 16 schools and two research institutions. The study findings may not be generalizable to other institutions. To make the study findings more representative, a large scale study is needed involving more academic and research institutions. The purpose of the current study is to gain insight on gender integration in science and research, describe female participation in science and research and identify the associated factors. Second, non-response may affect the external validity of the study. To mitigate this, we have allowed for a 10% non-response rate. Furthermore, limited access to research data from the two institutions may affect the quality of the study. Finally, the cross-sectional study will not allow conclusion on causality. A longitudinal study is needed to allow for conclusion causation of low female participation in science and research.

Despite these limitations, we believe that the study is likely going to contribute to the scientific body of knowledge on the integration of gender mainstreaming in science and research. The findings will also provide insight on female participation in science and research and the factors which affect their participation. Currently, no such study has been conducted in the country and information on this subject is scant

2.16. Costs and payments

There is no cash payment provided to participants for any portion of the study. Participants volunteer the time taken to complete this survey. As a small token of appreciation for their time and opportunity costs, IDI participants will receive a bottle of water or soft drink, in line with UNZABREC procedures.

2.17. Contributors

CS led the design the study, drafting of the study protocol and implementation of the study, and drafted this manuscript. JMZ and MPC contributed to the study protocol. PIS, NBM and NZ contributed to the development of the data collection instruments, enlisting the study sample, and coordinated data collection

For peer review only

process. PIS and NZ contributed to the revision of the manuscript. All authors read and approved the manuscript.

2.18. Funding

This work was supported by the National Science and Technology Council (grant number NSTC 101/6/8; **supplementary material 6**), as part of the Gender dimensions in science, technology and innovation under the strategic research fund. The funders had no influence on the design, implementation of the study and draft of this manuscript. The protocols underwent a rigorous an external peer review process (**Funder Peer Review**; **Response to Reviewer Comments**)

2.19. Conflict of interest

We declare that authors have no conflicting interest

References

1. UN Women (1985). World Conference to review and appraise the achievements of the United Nations Decade for Women: Equality, Development and Peace. Available at: http://www.un-documents.net/nflsaw.htm
2. World Conference on Science (1999). Science for the first twenty first Century. Available at: http://www.unesco.org/science/wcs/abstracts/II_4_gender.htm
3. United Nations (2015). Sustainable Development Goals: 17 Goals to transform our world. Available at: https://www.un.org/development/desa/disabilities/envision2030.html
4. United Nations Sustainable Development Group (2018). Gender equality and Empowerment of Women: UNCT-SWAP Gender Equality Scorecard. Available at: https://unsdg.un.org/resources/unct-swap-gender-equality-scorecard
5. United Nations (2015). Sustainable Development Goals. Transforming our world: the 2030 Agenda for Sustainable Development. Available at: https://sustainabledevelopment.un.org/post2015/transformingourworld

For peer review only

6. Ministry of Gender and Child Development (2014). National Gender Policy 2014. Available at: https://genderlinks.org.za/srhrs/zambia-national-gender-policy-2014/

7. UNESCO. Science, Technology and Innovation Policy. Available at: http://www.unesco.org/new/en/natural-sciences/science-technology/sti-systems-and-governance/

8. JICA (2016). Country Gender Profile: Zambia Final Report. Available at: https://www.jica.go.jp/english/our_work/thematic_issues/gender/background/c8h0vm0000anjqj6-att/zambia_2016.pdf

9. ASTI. African Women in Agricultural Research and Development. Available at: https://asti.cgiar.org/gender

10. Ministry of Gender and Child Development and Central Statistics Office. Gender Status report 2012-2014. Available: https://www.zamstats.gov.zm/phocadownload/Gender/Gender%20Status%20Report%202012-2014%20290616.pdf

11. European Commission (2016). Director General for Research and Innovation. Available at: https://ec.europa.eu/info/publications/directorate-general-research-and-innovation_en

12. Gender in Science Innovation, Innovation, Technology and Engineering. Available: https://genderinsite.net/about/who-we-are

13. UNESCO. Sustainable Development Goals for Natural Sciences. Available at: https://en.unesco.org/sustainabledevelopmentgoalsfornaturalsciences

14. UNESCO. UNESCO Science report: Towards 2030: Measuring progress towards Sustainable Development Goal 9, available at: https://en.unesco.org/unesco_science_report/sdg9

For peer review only

15. Castillo R, Grazzi M, & Tacsir E (2014). Women in Science and Technology. What Does the Literature Say? Inter-American Development Bank. Available at: https://publications.iadb.org/en/women-science-and-technology-what-does-literature-say

16. Lee, H. and Pollitzer, E., 2016. Gender in science and innovation as component of inclusive socioeconomic growth. Portia Limited.19 Nicia, G. and Luisa, S., 2020. A gender-based assessment of Science, Technology and Innovation ecosystem in Mozambique. African Journal of Rural Development, 5(1),

17. Lardies, C.A., Dryding, D. and Logan, C., 2019. Gains and Gaps: Perceptions and Experiences of Gender in Africa

18. Kunda, D., Chembe, C. and Mukupa, G., 2018. Factors that influence Zambian higher education lecturer's attitude towards integrating ICTs in teaching and research. *JOTSE*, *8*(4), pp.360-384

19. Miroux, A., 2011. Mainstreaming a gender perspective in science, technology and innovation policy. United Nations Commission on the Status of Women.

20. Nyanja, N. and Musonda, E., 2020. A review of the ICT subject implementation in schools: a perspective of Lusaka Province (Zambia). Education and Information Technologies, 25(2), pp.1109-1127.

21. Hamusankwa M. and Munsaka, E., Gendered Experiences of Female Engineering Students in Selected Public Universities in Zambia.

22. Kipsoi, E.J., Chang'ach, J.K. and Sang, H.C., 2012. Challenges facing adoption of information communication technology (ICT) in educational management in schools in Kenya. Journal of Sociological research, 3(1), pp.18-28.

23. Morsy H (2019). Mainstreaming gender in African policymaking: Ensuring no voice is unheard. Available at: https://reliefweb.int/report/world/mainstreaming-gender-african-policymaking-ensuring-no-voice-unheard

24. Zavale, N.C., 2018. Expansion versus contribution of higher education in Africa: University–industry linkages in Mozambique from companies' perspective. Science and Public Policy, 45(5), pp.645-660.

25. United Nations. Envision 2030 Goal 9: Industry, Innovation, and Infrastructure. Available at: https://www.un.org/development/desa/disabilities/envision2030-goal9.html

For peer review only

26. UNESCO. Gender equality guidelines for UNESCO publications. Available at: https://en.unesco.org/system/files/ge_guidelines_for_publications_-_annex_5.pdf
27. UN Women. Gender mainstreaming. Available at: https://www.un.org/womenwatch/osagi/gendermainstreaming.htm
28. UNICEF (2007). Gender main streaming Assessment. Available at: https://www.unicef.org/evaldatabase/files/EO_2007_Gender_Mainstreaming_Report.pdf
29. King N and Horrocks C. 2010. Interviews in Qualitative Research. 1st Edition. SAGE Publications ltd. 25-41.
30. Ary D, Jacobs L, Sorensen C and Walker D. Introduction to research in education. 9th edition. Wadsworth, CA: Cengage Learning, 2013
31. Whitley W and Ball J (2002). Statistics review 4: Sample size calculations. Available online: Available online http://ccforum.com/content/6/4/335

For peer review only

Table 1: IDI participant distribution per institution

Participant	UNZA	CBU	Mount Makulu	TDRC	Total
Registrar	1	1			2
Head of institution	0	0	1	1	2
Deans	4	4	0	0	6
Heads of Department	4	4	2	2	14
Lecturer	3	3	0	0	6
Total	12	12	3	3	30

For peer review only

QUESTIONNAIRE FOR ACADEMICIANS & RESEARCHERS

Exploring the gender dimensions and factors affecting female participation in Science and Health Research in Zambia

QUESTIONNAIRE ID……………………………………………………………………………………

NAME OF INSTITUTION: ………………………………………………………………………………

SCHOOL: ……………………………………………………………………………………………

DEPARTMENT: ………………………………………………………………………………………

Summary of the study

For peer review only - http://bmjopen.bmj.com/site/about/guidelines.xhtml

Despite the Zambian government making remarkable progress on prioritizing gender mainstreaming, female participation in science, technology and innovation (STI) in academia, research and development is still low. Furthermore, information on the integration of gender dimension and the factors affecting inclusion of men and women in STI in research & development is scant. The aim of this research project is to determine the integration of gender dimensions and the factors that influence female participation in scientific and health research in the country in order to bridge the knowledge gaps and inform policy and programming to support the advancement of gender equality in STI in the country. To this end, the National Science Technology Council has funded this study.

Instructions

You are one of the 400 participants requested to take this questionnaire. Please read carefully and answer all the questions. This questionnaire (60 questions) seeks to collect information on your opinion and perspectives on the extent to which females are involved in and the factors influencing female participation in science-related fields of study in Zambia.

1. Age in years: ……………………………………………..

For peer review only - http://bmjopen.bmj.com/site/about/guidelines.xhtml

2. Sex:
    1. Male
    2. Female

3. Marital status:
    1. Single
    2. Married
    3. Divorced
    4. Widow
    5. Cohabiting

4. If married, is your partner currently employed?
    1. Yes
    2. No
    3. Not applicable (select this option if not married)

5. Do you have children?
    1. Yes
    2. No

6. If yes, indicate how many (actual number): ……………………

7. Field of Study (select one most appropriate):
    1. Natural Sciences
    2. Mining
    3. Engineering
    4. Medicine
    5. Veterinary Medicine
    6. Public Health
    7. Health Sciences
    8. Nursing
    9. Others (specify) :…………………

8. Have you always considered a career in this field?
    1. Yes
    2. No

9. Who influenced your choice of the career?
    1. Personal interest
    2. Father
    3. Mother
    4. Sibling
    5. Family member
    6. Friend
    7. Teacher
    8. Career counsellor
    9. Others (specify)……………………………………………..

For peer review only - http://bmjopen.bmj.com/site/about/guidelines.xhtml

10. Why did you enroll in this program initially?
    1. Interest in the subject matter
    2. Field in which I excelled
    3. Influence of faculty members
    4. Influence of advisor or mentor
    5. Prospects for personal development
    6. Social aspect of expected job
    7. Work conditions and benefits expected
    8. I always thought I would study in this field
    9. Other, please specify:……………….

10. Indicate your highest academic qualification:
    1. Diploma
    2. BSc/BA
    3. MSc/MA
    4. PhD
    5. Postdoc

11. Which of the following best describes your current status?
    1. Currently working in an STI academic institution
    2. Currently working in a research institution

12. Employment status:
    1. Permanent and Pensionable
    2. Contractual

13. Indicate the number of years working in this institution…..……….

14. Indicate your current position:
    1. Staff development fellow;
    2. Lecturer 1
    3. Lecturer 2
    4. Lecturer 3
    5. Associate Professor Researcher
    6. Full Professor
    7. Administrative staff

15. Indicate the number of years in this position…...……………..

16. From your personal point of view, which one of the following factors were important in obtaining your current position?
    1. Earlier cooperation with faculty members of your current work place
    2. Personal contacts at your current work place previous to obtaining your position
    3. A network of people who could testify to your professional qualifications
    4. Programs promoting gender parity at work
    5. Expansion of higher education sector
    6. Formal or informal professional experience outside the academic sector
    7. Number of publications or patents
    8. Number of grants awarded

For peer review only - http://bmjopen.bmj.com/site/about/guidelines.xhtml

17. Job satisfaction:
    1. Yes;
    2. No;
    3. I am not sure

18. Have you been involved in Science and academic research?
    1. Yes
    2. No

If the answer is No, indicate "Not applicable" on questions 19, 20 and 21, and proceed to question 22.

19. If the answer is yes, at what level have you been involved?
    1. Research Assistant
    2. Field Supervisor
    3. Collaborator
    4. Co-investigator
    5. Principal investigator
    6. Not applicable

20. If the answer is yes, indicate number of research projects in past 2 years:
    1. 1-2
    2. 2-3
    3. 3-4
    4. 5-6
    5. >6
    6. Not applicable

21. Indicate reasons for involvement in research:
    1. Requirement for certification
    2. Academic promotion
    3. Contribution to knowledge
    4. For international recognition
    5. Not applicable

22. If the answer is no, indicate the reason:
    1. Limited skills in research;
    2. Limited access to research funds;
    3. Restrictive work policy;
    4. Not interested in research;
    5. Other (specify)…………

23. Are you currently seeking a new job?
    1. Yes
    2. No

24. If yes, why?

For peer review only - http://bmjopen.bmj.com/site/about/guidelines.xhtml

For peer review only

1. Better salary
2. Closer to family
3. Better work environment
4. More suitable job to my training & experience
5. Other (specify)……………..

25. Have you ever left a job in your field?
    1. Yes
    2. No

26. If yes, why?
    1. You were harassed at work
    2. You knew of someone who was harassed at work
    3. You were offered a better job
    4. You found a better new job
    5. Others, Specify………..

27. Do you consider yourself to have enough opportunities to participate in research projects?
    1. Yes
    2. No

28. If yes, mention which ones
    1. Locally funded research projects
    2. Internationally funded research projects
    3. Both locally and internationally funded research projects
    4. Not applicable (***select this option if your response to question 27 is*** "**No**")

29. If the answer is no, mention why?
    1. Did not receive any funding
    2. Did not receive any information on funding opportunities
    3. Could not find information on funding opportunities
    4. Was not authorized to apply for research funds
    5. Did not look into how to access to funding opportunities
    6. Other, specify…….

30. Do you consider yourself to have enough opportunities to participate in conferences and workshops?
    1. Yes
    2. No

31. If yes, mention which ones?
    1. Local conferences and workshops
    2. International conferences and workshops
    3. Both local and international conferences and workshops
    4. Not applicable (***select this option if the response to question 30 is "No"***)

32. If the answer is no, mention why?
    1. Did not receive any funding
    2. Did not receive any information on opportunities
    3. Could not find information on opportunity

For peer review only - http://bmjopen.bmj.com/site/about/guidelines.xhtml

4. Was not authorized
5. Did not look into how to access such opportunities
6. Other, specify…….

Which of the following policies is implemented at the organization where you are employed

Question	1. Yes	2. No	3. Don't know
33. Gender Equality Plan			
34. Code of Conduct			
35. Office for investigating complains			

Does the organization you work for have the following career policies and practices in place?

Question	1.Yes	2.No	3.Don't know
36. Gender parity in promotion			
37. Personal development training			
38. Career mentoring			
39. Flexible working hours			
40. Telework/remote with policies and guidelines			
41. Telework/remote with policies and guidelines			

42. Have you ever had a period of time during your professional training or in your career where it was difficult for you to fulfill your professional responsibilities because of your personal responsibilities?
    1. Yes
    2. No

43. If yes, what were the main reasons?
    1. Care for children
    2. Care for family member (other than children)
    3. Illness/accident
    4. Burn out
    5. Depression
    6. Change in a marital situation (for example divorce)
    7. Death in family or of closely related person
    8. Other, specify……………
    9. Not applicable (**select this option if your answer to question 42 is "No"**)

44. Do you have dependants (children or other persons under your responsibility)?
    1. Yes
    2. No
45. If yes, how many dependants are in your care? Indicate actual number: ……………...

For peer review only - http://bmjopen.bmj.com/site/about/guidelines.xhtml

For peer review only

46. Age of dependants:
    1. ___________ between 0 and 5 years
    2. ___________ between 5 and 18 years
    3. ___________ between 19 and 65 years
    4. ___________ 65 years and over

47. Have you ever received any financial incentives for your accomplishments?
    1. Yes
    2. NO

48. Have you ever received any non-financial incentives for your accomplishments?
    1. Yes
    2. NO

49. Have you ever received any awards for your accomplishments?
    1. Yes
    2. NO

50. Have you ever been invited to participate in committees by the Ministry responsible for Science, Technology and Innovation (STI) in Zambia
    1. Yes
    2. No

51. Have you applied for a research grants before?
    1. Yes
    2. No

52. If yes, how many times have you applied for grants from national and international institutions over the past 2 years? Indicate number:…………….

53. If yes, what was your role?
    1. Principal investigator
    2. Part of a team

54. Where did you apply?
    1. National funding institution
    2. International funding institution
    3. Both national and international funding institution

55. How many national grants have you won? Indicate number……………………...

56. How many international grants have you won? Indicate number………..……….

57. Can women be as successful as men in STI in academia, Industry-research and development?
    1. Yes

For peer review only - http://bmjopen.bmj.com/site/about/guidelines.xhtml

2. No

58. Do you think your field or job is equally suited for both sexes?
    1. More suitable for men
    2. More suitable for women
    3. Suitable for both men and women

59. Are you aware of instances of a pay gap between men and women who have the same responsibilities in your organization?
    1. Yes
    2. No

60. How frequently do you engage in presentation to the public at a science center?
    1. Never
    2. Rarely
    3. Sometimes
    4. Often
    5. Very often

THE END

For peer review only

For peer review only - http://bmjopen.bmj.com/site/about/guidelines.xhtml

INTERVIEW GUIDE FOR ACADEMIC INSTITUTIONS

Gender dimensions and factors affecting female participation in Science and Health Research in Zambia

Theme 1: Policies on Integration of Gender Dimension in scientific and health research

Sub-theme 1.1: Policies on student recruitment

Probe for:

- Available policies on student recruitment in scientific disciplines at undergraduate level
- Available policies on student recruitment in scientific disciplines at postgraduate level
- Dissemination of available policies in the institution
- Extent to which available policies are implemented in the institution
- Policy implementation with regard to promotion of academic staff

Sub-theme 1.2: Policies on staff recruitment and promotion

Probe for:

- Available policies on staff recruitment and promotion in the institution
- Integration of gender in the recruitment and promotion of staff to academic positions
- Integration of gender in the recruitment and promotion of staff to administrative position
- Dissemination of available policies in the institution
- Extent to which available policies are implemented in the institution

Sub-theme 1.3: Policies on Integration of Gender Dimension in Scientific and health research

Probe for:

- Available policies on scientific research and innovation in the institution (eg policy on student recruitment at undergraduate, postgraduate, mention available policies)
- Integration of gender in the available policies
- Mention specific aspects of gender integration in the policies
- Extent to which the available policies are disseminated in the institution
- Extent to which the available policies are implemented in the institution

For peer review only

Theme 2: Participation in Scientific and Health Research

Probe for:

- Extent to which staff in the institution are involved in Scientific and research n
- Extent to which gender dimension is considered in staff participation in scientific and research
- (opportunities for research grants; award of internal and external grants, attending local and international conferences)
- Extent to which gender dimension is considered in staff career path in science and research
- Extent to which gender dimension is considered in staff promotion in scientific and health research
- Suggestions for improving gender integration and female participation in scientific and health research

Theme 3: Factors influencing gender integration and female participation in scientific and health research

Probe for both facilitators and barriers:

- Institutional norms and prejudices on female participation and gender integration in Scientific and health research
- Decision-making on award of research grants in the institution
- Attitude towards female participation in scientific and health research
- Structural factors in the institution (eg, workload, hierarchy)
- Family and personal factors (marriage, religious)

For peer review only

INTERVIEW GUIDE FOR RESAERCH INSTITUTIONS

Exploring the gender dimensions and factors affecting female participation in scientific and health research in Zambia

Theme 1: Policies on Integration of Gender Dimension in scientific and health research

Sub-theme 1.1: Policies on staff recruitment and promotion

Probe for:

- Available policies on staff recruitment and promotion in the institution
- Integration of gender in the recruitment and promotion of staff to scientific and
- Integration of gender in the recruitment and promotion of staff to administrative position
- Dissemination of available policies in the institution
- Extent to which available policies are implemented in the institution

Sub-theme 1.2: Policies on Integration of Gender Dimension in Scientific and health research

Probe for:

- Available policies on scientific research and innovation in the institution (eg policy on recruitment mention available policies)
- Integration of gender in the available policies
- Mention specific aspects of gender integration in the policies
- Extent to which the available policies are disseminated in the institution
- Extent to which the available policies are implemented in the institution

Theme 2: Participation in Scientific and Research

Probe for:

- Extent to which staff in the institution are involved in Scientific research and Innovation
- Extent to which gender dimension is considered in scientific research and Innovation and grant opportunities (award of internal and external grants, attending local and international conferences)
- Extent to which gender dimension is considered in staff career path in Scientific research and Innovation (training opportunities, etc)

For peer review only

- Extent to which gender dimension is considered in staff promotion
- Suggestions for improving gender integration and female participation in the institutions

Theme 3: Factors influencing gender integration and female participation in scientific and health research

Probe for both facilitators and barriers:

- Institutional norms and prejudices on female participation and gender integration in Scientific and health research
- Decision-making on award of research grants in the institution
- Attitude towards female participation in STI in academia and research
- Structural factors in the institution (eg, workload, hierarchy)
- Family and personal factors (marriage, religious)

For peer review only

A. Research Grants: Local Funding (source within Zambia)

Name of the School:……………………………………………

Year	2017		2018		2019		2020		2021		Total		Grand Total
	M	F	M	F	M	F	M	F	M	F	M	F	
SDF													
Lecturer 3													
Lecturer 2													
Lecturer 1													
Associate Professor													
Full Professor													
Total for the year													

B. Research Grants: International Funding (Foreign Source)

Name of the School:……………………………………………

Year	2017		2018		2019		2020		2021		Total		Grand Total
	M	F	M	F	M	F	M	F	M	F	M	F	
SDF													
Lecturer 3													
Lecturer 2													
Lecturer 1													
Associate Professor													
Full Professor													
Total for the year													

C. Publications in international Peer Reviewed Journals

Name of the School:……………………………………………

Year	2017		2018		2019		2020		2021		Total		Grand Total
	M	F	M	F	M	F	M	F	M	F	M	F	
SDF													
Lecturer 3													
Lecturer 2													
Lecturer 1													
Associate Professor													
Full Professor													
Total for the year													

For peer review only

For peer review only

Table 2: Summary of data collection techniques

Objective	Technique	Target institution/ group	Instrument
Explore gender integration in science and research	Desk and literature review	UNZA<br>Copperbelt University (CBU)<br>Ministry of Higher Education<br>Ministry of Gender	Checklist
Perspective on factors affecting female participation in science and research	IDI	UNZA<br>CBU<br>Mount Makulu<br>TDRC	Interview guide
Identify the factors that affect female participation in science and research	Survey	UNZA<br>CBU	Questionnaire

For peer review only

UNIVERSITY OF ZAMBIA
BIOMEDICAL RESEARCH ETHICS COMMITTEE

Telephone: 260-1-256067
Telegrams: UNZA, LUSAKA
Telex: UNZALU ZA 44370
Fax: + 260-1-250753
Federal Assurance No. FWA00000338

Ridgeway Campus
P.O. Box 50110
Lusaka, Zambia
E-mail: unzarec@unza.zm
IRB00001131 of IORG0000774

10th May 2021

Your REF. No. 1674-2021

Dr. Cephas Sialubanje,
Levy Mwanawasa Medical University,
School of Public Health,
Department of Health Promotion and Disease Prevention
P.O Box 33991,
Lusaka.

Dear Dr. Sialubanje,

RE: EXPLORING THE GENDER DIMENSIONS AND FACTORS AFFECTING FEMALE PARTICIPATION IN SCIENCE, TECHNOLOGY AND INNOVATION (STI) IN ACADEMIA, INDUSTRY-RESEARCH & DEVELOPMENT IN ZAMBIA (REF. NO. 1674-2021)

The above-mentioned research proposal was presented to the Biomedical Research Ethics Committee on 7th May, 2021. The proposal is **approved**. The approval is based on the following documents that were submitted for review:

a) Study proposal
b) Questionnaires
c) Participant Consent Form

APPROVAL NUMBER : REF. 1674-2021

This number should be used on all correspondence, consent forms and documents as appropriate.

- **APPROVAL DATE : 10th May 2021**
- **TYPE OF APPROVAL : Fast Track**
- **EXPIRATION DATE OF APPROVAL : 9th May 2022**
  After this date, this project may only continue upon renewal. For purposes of renewal, a progress report on a standard form obtainable from the UNZABREC Offices should be submitted one month before the expiration date for continuing review.
- **SERIOUS ADVERSE EVENT REPORTING**: All SAEs and any other serious challenges/problems having to do with participant welfare, participant safety and study integrity must be reported to UNZABREC within 3 working days using standard forms obtainable from UNZABREC.

For peer review only

- **MODIFICATIONS**: Prior UNZABREC approval using standard forms obtainable from the UNZABREC Offices is required before implementing any changes in the Protocol (including changes in the consent documents).
- **TERMINATION OF STUDY**: On termination of a study, a report must be submitted to the UNZABREC using standard forms obtainable from the UNZABREC Offices.
- **NHRA:** You are advised to obtain final study clearance and approval to conduct research in Zambia from the National Health Research Authority (NHRA) before commencing the research project.
- **QUESTIONS**: Please contact the UNZABREC on Telephone No.256067 or by e-mail on unzarec@unza.zm.
- **OTHER**: Please be reminded to send in copies of your research findings/results for our records. You're also required to submit electronic copies of your publications in peer-reviewed journals that may emanate from this study. Use the online portal: unza.rhinno.net for further submissions.

Yours sincerely,

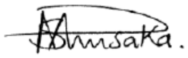

Sody Mweetwa Munsaka, BSc., MSc., PhD
CHAIRPERSON
Tel: +260977925304
E-mail: s.munsaka@unza.zm

For peer review only

STROBE Statement—Checklist of items that should be included in reports of cohort studies

**Item**	**Recommendation**	Page
Title and abstract	Indicate the study's design with a commonly used term in the title or the abstract	1
	Provide in the abstract an informative and balanced summary of what was done and what was found	2
Introduction		
Background/rationale	Explain the scientific background and rationale for the investigation being reported	4
Objectives	State specific objectives, including any pre-specified hypotheses	5
Methods		
Study design	Present key elements of study design early in the paper	6
Setting	Describe the setting, locations, and relevant dates, including periods of recruitment, exposure, follow-up, and data collection	6
Participants	Give the eligibility criteria, and the sources and methods of selection of participants. Describe methods of follow-up	7
Study size	Explain how the study size was arrived at	8
Variables	Clearly define all outcomes, exposures, predictors, potential confounders, and effect modifiers. Give diagnostic criteria, if applicable	9
Data sources/ measurement	For each variable of interest, give sources of data and details of methods of assessment (measurement). Describe comparability of assessment methods if there is more than one group	9
Bias	Describe any efforts to address potential sources of bias	11
Statistical methods	Describe all statistical methods, including those used to control for confounding	12
	If applicable, explain how loss to follow-up was addressed	
Patient and public involvement		12
Ethical considerations		13
Limitations		14
Funding		15

For peer review only

Gender integration and female participation in scientific and health research in Zambia: A descriptive cross-sectional study protocol

Journal:	*BMJ Open*
Manuscript ID	bmjopen-2022-064139.R1
Article Type:	Protocol
Date Submitted by the Author:	01-Feb-2023
Complete List of Authors:	Sialubanje, Cephas; Levy Mwanawasa Medical University, Levy Mwanawasa Medical University<br>Sumbwa, Phyllis; University of Zambia<br>Zulu, Nyondwa; University of Zambia, School of Education<br>Mwanza, Nchimunya; University of Zambia, School of Public Health<br>Chavula, malizgani; University of Zambia, School of Public Health<br>Zulu, Joseph; University of Zambia, School of Public Health
**Primary Subject Heading**:	Public health
Secondary Subject Heading:	Health services research, Qualitative research, Research methods, Epidemiology
Keywords:	HEALTH SERVICES ADMINISTRATION & MANAGEMENT, Health policy < HEALTH SERVICES ADMINISTRATION & MANAGEMENT, Human resource management < HEALTH SERVICES ADMINISTRATION & MANAGEMENT

SCHOLARONE™ Manuscripts

Gender integration and female participation in scientific and health research in Zambia: A descriptive cross-sectional study protocol

Cephas Sialubanje[1], Phyllis Ingutu Sumbwa[2], Nyondwa Zulu[2], Nchimunya B. Mwanza[3], Malizgani Paul Chavula[3], Joseph M. Zulu[3]

[1]School of Public Health, Levy Mwanawasa Medical University, P.O. Box 33991, Lusaka, Zambia

[2] School of Education, University of Zambia, P.O. Box 323791, Lusaka, Zambia

[3]School of Public Health, University of Zambia, P.O. Box 32379, Lusaka, Zambia

For peer review only

For peer review only - http://bmjopen.bmj.com/site/about/guidelines.xhtml

Abstract

Introduction: Despite the Zambian government making progress on prioritizing gender mainstreaming, female participation in science, technology and innovation in academia, research and development is still low. This study aims to determine the integration of gender dimensions and the factors that influence female participation in science and health research in Zambia.

Methods and analysis: We propose a descriptive cross-sectional study design employing in-depth interviews (IDIs) and survey as data collection techniques. Twenty schools offering science-based programs will be purposively selected from the university of Zambia (UNZA), Copperbelt, Mulungushi and Kwame Nkrumah Universities. In addition, two research institutions, Tropical Disease Research Centre and Mount Makulu Agricultural Research station will be included. Survey respondents will include a random sample of 1,389 academic and research staff from the selected schools. A total of 30 IDIs will be conducted with staff and heads of selected schools and research institutions. Data collection will be conducted over a 12 month period. Before commencement of data collection, in-depth literature search and record review on gender dimensions in science and health research will be conducted to gain insight into the subject matter and inform research instrument design. Survey data and IDIs will be collected using a structured paper-based questionnaire and semi structured interview guide, respectively. Descriptive statistics will be computed to summarise respondents' characteristics. Bivariate analysis (chi square and independent t-test) and multivariate regression analysis will be conducted to test the association and identify factors influencing female participation in science and health research (adjusted odds ratios, $p<0.05$). An inductive approach will be used to analyse qualitative data using N-vivo. Survey and IDI will be corroborated.

Ethics and dissemination: Ethical approval was obtained from UNZA Biomedical Research Ethics Committee. Study findings will be disseminated through a written report, stakeholder meetings and publication in a peer reviewed international journal.

Word count: 298

Key words: gender mainstreaming, gender discrimination, gender equality, Zambia, representation. Science, Health research, sustainable development goals

For peer review only - http://bmjopen.bmj.com/site/about/guidelines.xhtml

Strengths and limitations of the study

- Use of mixed methods cross-sectional design employing in-depth literature review, survey and in-depth interview techniques increases internal validity of the study
- Use of probability proportional to size (PPS) and random sampling techniques to select schools and study participants, respectively, minimizes bias due to selection bias
- Training of data collectors minimizes measurement error
- Cross-sectional design of the study does not allow for causal inference
- Selection of all the 16 schools offering science-based programs from only two public institutions in may introduce selection bias.

For peer review only

For peer review only - http://bmjopen.bmj.com/site/about/guidelines.xhtml

1. Introduction
1.1. Background

Gender mainstreaming is a public policy concept of assessing different implications of any public policy action, legislation and programs on people of different genders [1]. The concept was first proposed at the 1985 third World Conference on Women in Nairobi, Kenya [1]. It has been adopted into the United Nations' (UN's) development community as a globally accepted strategy for promoting gender equality [2] and ensuring that all UN member states achieve the 50 percent gender parity−through empowering all women and girls, ending all forms of discrimination against women and girls everywhere and ensuring women's full and effective participation and equal opportunities at all levels of decision making [3]. Gender equality ensures that women have the same opportunities in life as men, including the ability to participate in the public sphere [4]. It also ensures that gender perspectives and goals are central to all activities including policy development, research, advocacy/ dialogue, legislation, resource allocation, planning, implementation and monitoring of programmes and projects. Gender equality also ensures equitable distribution of benefits from the development process for boys, girls, men and women. It is at the heart of strategies and interventions driving the active and meaningful participation of both women and men in the development agenda and ensures consistent empowerment of women and girls [4]. It is a basic requirement for measuring progress on sustainable development goals (SDGs) and constitutes a key aspect within the principle of leaving no one behind, without which the SDGs cannot be achieved [5]. Both gender equality and women's empowerment are integral to realizing the 2030 Agenda for sustainable development [6] and all the UN SGDs [6] which were agreed upon in the UN Country Team (UNCT) System-wide Action Plan (SWAP). Thus, the gender dimension of science and technology (S&T) has become an increasingly important and topical issue worldwide.

Globally, governments are increasingly recognizing the importance of supporting the study of science and technology at tertiary levels including vocational and technical training, undergraduate and graduate education of women and men with interest, talents and capabilities lie in the fields [7]. Consequently, enrolment of women at the tertiary level has increased over the past few decades, with women now approaching 50% of the total number of tertiary students worldwide. The greatest gains in absolute terms have been made in developing countries. However, there are wide variations at the country and region levels [7].

For peer review only - http://bmjopen.bmj.com/site/about/guidelines.xhtml

Nevertheless, women and girls around the world are still excluded from participation in science and technology and gaps remain in the area of women researchers and scientist in top managerial positions throughout the world [7-9]. In Southern Africa, less than 30% of women participate in research; the proportion variations across the countries, ranging from 8.7% in Congo DR to 44.9% in South Africa and 47% in Eswatini. The most preferred areas of research focus are natural sciences (31%) and agricultural and veterinary sciences (18.7%). Although the European Union for Research and Innovation (2022) report [10] shows that 57% of the EU tertiary graduates are women, less than 4% of female students take degrees in engineering, manufacturing, construction and ICT which are predominantly chosen by males; only 23% of women participate in medical science research, 19% in natural sciences research and 4% in agricultural sciences research.

Zambia is one of the UN member states that have adopted the gender mainstreaming concept and developed a national gender policy [11]. The policy focuses on attainment of gender equality in the development process and providing for equal opportunities for women and men to actively participate and contribute to their fullest ability. It also focuses on equitable benefits from national development.

Although the Zambian government has made significant progress in adopting the policy [12] and increasing female enrolment in secondary and higher education as well as their representation in decision-making positions [13-15], female representation and participation in science, technology and innovation (STI) is still low and far from achieving the UN 50% gender parity target. Reports and studies conducted in Zambia [13-15] reveal unequal gender representation in STI with women continuing to lag behind their male counterparts; their access and retention in tertiary education remains a challenge. For example, the Ministry of Gender and Child Development report [15] shows that females only comprised 39.1% of the combined total enrollment in the four Zambian Public Universities (UNZA, Copperbelt, Mulungushi and Nkrumah).

Reasons for the low female participation in this field are not well understood [16-19]. Several factors including poverty, lack of education and aspects of their legal, institutional, political and cultural environments have been shown to affect women from participating in science and innovation. However, there is dearth of published literature on this subject; most published studies relate to developed countries and regions such as the United States and Europe [20]. The few published studies focus on information, communication and technology (ICT) [21-25] in secondary and higher institutions of learning, which are not applicable to STI in academia, research and development. Thus, the actual gender context with regard to integration of gender dynamics and the issues affecting female participation in science and health research in the country are not

For peer review only - http://bmjopen.bmj.com/site/about/guidelines.xhtml

well understood despite the growing recognition of the importance of gender equality in this area. A study is therefore required to answer the following questions in order to bridge the knowledge gaps on this subject:

1) To what extent is gender mainstreaming integrated in science and health research?
2) What is the proportion of females participating in science and health research in Zambia?
3) What factors influence female participation in science and health research in the country?

1.2. Objectives

This study aims to explore the integration of gender dimensions and determine the predictors of female participation in scientific and health research in the country. The specific objectives are:

1) Explore integration of gender policy in academic and research institutions.
2) Explore female participation in the science and health research activities
3) Identify the factors that affect female participation in science and health research and development
4) Determine the predictors of female participation in scientific and health research

This knowledge is important for informing policy and programming for the advancement of gender equality, equal opportunities and participation in science, health research and development in order to promote quality scientific and health outcomes and technological excellence. Incorporating gender dimensions and deliberately encouraging females to participate in science and health research is crucial in realizing the key role that females can play in the development and advancement of science, and their commitment to inclusive science and research development. Gender equality in science and research is not only a matter of fairness but important for enhancing the recruitment of the most talented individuals, irrespective of gender, and for tapping a partially unexploited resource [26,27]. It is a way to promote scientific and technological excellence rather than just improving opportunities for women [28, 29]. A more inclusive workforce is likely to be more innovative and productive than one which is less so [30]. Moreover, having scientists and researchers with diverse backgrounds, interests, and cultures assures better scientific research and the best use of those results [31]. Mainstreaming gender in science and health research is crucially important for various reasons. First, women are the main driving labor force in most African countries, making a vital contribution to the economy. Empirical evidence [32] shows that if women had equal access to productive resources such as science, technology, research, or financial services, the gender productivity gap would almost disappear and

For peer review only - http://bmjopen.bmj.com/site/about/guidelines.xhtml

African countries, including Zambia, could significantly increase their production by just closing the gender gap across economic, academic and scientific divide. Second, increasing women's participation in STI has the potential to lead to higher GDP growth through increases in higher-skilled women in the labor force and women's employment in high-productivity sectors. Gender equality and empowerment of females to participate in science and health research can also boost women's role as leaders in the field [32, 33].

2. Methods

2.1. Study design

We propose to conduct a descriptive mixed methods cross-sectional study design employing in-depth interviews (IDIs) and survey as data collection techniques. Use of qualitative and quantitative data collection and analysis techniques will make the study comprehensive and allow for triangulation and corroboration of the findings. This will in turn increase the internal validity of the findings [34,35]

2.2. Study setting

The study will be conducted at four public institutions of higher learning in Zambia (university of Zambia ((UNZA), Copperbelt, Kwame Nkrumah and Mulungushi universities) and two public research institutions (Tropical Disease Research Centre ((TDRC) and Mount Makulu Research Centre. In addition, one Science Granting Council, Higher Education Loans and Scholarship Board (HESB), two government ministries (Gender and Higher Education) and Un agencies (UNICEF and UNESCO) will be included in the study. UNZA is located in Lusaka, the capital city of Zambia. It is the largest and oldest public university in the country. It was founded in 1965 and established by the Act of Parliament No. 66 of 1965. The first intake of students took place on 17th March 1966. It offers a number of academic programs at diploma, undergraduate and postgraduate levels. The university has 13 faculties including Agricultural Sciences, Education, Engineering, Graduate School of Business, Health Sciences, Law, Medicine, Mining, Public Health, Natural Sciences, Nursing Sciences, Social Sciences and Humanities, Veterinary Medicine. It also has the directorate of research and graduate studies. The Copperbelt University is the second largest and oldest public university. It is located in Kitwe, Zambia and has 12 schools including: School of the Built Environment, School of Business, Dag Hammarskjold Institute for Peace and Conflict Resolution, Directorate of Distance Education and Open Learning, School of Engineering, School of Graduate studies, School of Mathematics and Natural Science, School of Medicine, School of Mines and Mineral Resources, School of Natural Resources, School of Information and Communication Technology, and School of Humanities. Mulungushi and Kwame Nkrumah universities are located in Kabwe, the headquarters for central province. TDRC is a public health research

For peer review only - http://bmjopen.bmj.com/site/about/guidelines.xhtml

institution located in Ndola, Copperbelt province. Mount Makulu is a public agricultural research institution located in Chilanga district, Lusaka province—approximately 20 kilometers from the national capital, Lusaka. HESB is a public institution located in Lusaka. It provides loans and scholarships to students in to pursue higher education both in Zambia and outside.

A total of sixteen schools offering science and health-related programs will be purposively selected from the selected institutions. Ten schools offering science- and health-related programs will be purposively selected from UNZA, namely: Medicine, Public Health, Nursing Sciences, Health Sciences, Veterinary Medicine, Mines, Engineering, Natural Sciences, Agricultural Sciences, Directorate of research and Postgraduate studies. Seven schools offering science and health-related programs at the Copperbelt university will be included in the study: School of Engineering, School of Mathematics and Natural Science, School of Medicine, School of Mines and Mineral Resources, School of Natural Resources, School of Information and Communication Technology and School of Graduate studies. In addition, two schools from Mulungushi and one from Nkrumah university will be included in the study. Two research institutions, TDRC from Ndola Zambia, and Mount Makulu Agricultural Research station in Chilanga will be included in the study.

2.3. Participants and sampling process

Survey participants will comprise a systematically selected sample of 1, 89 male and female academic and research staff from the selected schools . The academic and research institutions, science granting council and scholarship board have been selected because they are public and involved in science and health research.

To be eligible, survey participants will be:

- Lecturers and researchers at any academic rank (lecturer three to full professor)
- Having worked in the selected institution for at least 3 months

IDI participants will include staff from the selected schools and research institutions. To be eligible, IDI participants will be:

- Administrative staff from academic institutions (Registrar or Assistant Registra, School Dean or Assistant Dean, Head of Department or Section Head, director of directorate of research and graduate studies (DRGS)

For peer review only - http://bmjopen.bmj.com/site/about/guidelines.xhtml

- Administrative staff from research institutions (Head or assistant head of selected research institution, section head of research institution)
- Academic staff in all ranks
- Technical staff from research institutions

Survey participants will be selected using a stratified sampling technique. First, the number of staff from each school will be obtained. To ensure that all schools are proportionately represented, probability proportional to size (PPS) will be conducted to determine how many participants will be selected from each school. Next, a list of all the lecturers in each selected school will be compiled to serve as a sampling frame. To ensure inclusion of both male and female participants from in various academic and adminstrative ranks and that they have an equal chance of being selected, information on the sex (male and female) of potential respondnets will be obtained from the administrative managers and separate lists will be prepared for each sex. The sampling frame will also include other variables: qualification (first degree, masters and PhD), postion and rank in the organisation (from lowest rank to the full professor), administrative rank (ordinary lecturer, unit head, department health, Dean, Registrat, etc). In order to be comprehensive, the checlist will be prpeared by the research team in consulttaion with the administrative staff from the respective organisation.Each potential participant will be assigned a unique number on the sampling frame. A random number generator will be used to select participants from the list. The process will be done for each institution and school.

Purposive sampling will be used to recruit IDI participants from the selected institutions. In order to compare and contrast the respondents' views, both male and female participants will be included. We plan to conduct a total of thirty IDIs in both academic and research institutions. However, the actual number of IDIs to be conducted will be determined by the point of saturation [36-38].

In order to get variability in responses, compare and contrast the similarities and differences in participant perspectives on the subject, efforts will be made to ensure that both male and female respondents are included in the IDIs and questionnaire survey. Efforts will also be made to have a mix of survey participants from the lowest (lecturer 3) to the highest rank (Full professor).

2.4. Sample size estimation

In order to detect the difference in participation in scientific and health research between females and males, we will use the power approach to estimate the sample size. The power approach is used to

For peer review only - http://bmjopen.bmj.com/site/about/guidelines.xhtml

compute the sample required to detrmine the minimum difference between two or more groups in comparative studies [39,40]. In order to use this approach, the following assmuptions will be made:

- Power=80%
- Confience Interval 95% Confidence int
- Alpha=0.05
- Groups=2 (male and female)
- Sampling ratio=1
- Minimum detectable difference=15.0%
- Margin of error=5%

Inputting these assumptions into the online advanced power and sample size calculator [41] gives a mimim sample size of **604** per group or 1, 208 for the total sample. Allowing for a 15% non-response rate (NRR), gives a total sample size of **1,389 (or 695 for each group) survey respondents** required to have enough power to measure the proportion of females participating in STI and carry out multivariate analysis to determine the predictors of female participation in STI with 95% confidence.

2.5. Variables

The study variables will include the outcome and independent variables as follows:

Outcome variable: Female participation in science and health research and development

Independent variables:

a) Demographic information: age, sex, number of children
b) Field of study
c) Academic rank
d) Attitude towards scientific and health related research
e) Treatment at the workplace
f) Workplace culture

2.6. Data sources and collection techniques

In order to gain insight into the gender integration issues and inform design of data collection instruments, we propose to review of existing documents and policies from the UN agencies, government Ministries of

For peer review only - http://bmjopen.bmj.com/site/about/guidelines.xhtml

Gender and Higher Education, HESB, Science Granting Councils, academic and research institutions. The proposed search will include: a) enrolment and graduation data from the four selected academic institutions, b) administrative data on staffing profile in the selected schools; c) data on both external and internal research grants in the academic institutions, d) data on local grants from Science Granting Councils, e) review of national and institutional documents on gender policy and integration.

To determine the factors influencing gender integration and female participation in science and health research, a survey using a structured questionnaires will be conducted among a randomly selected sample of 1, 389 academic staff from the selected schools. To qualitatively explore the respondents' perspectives and gain insight into the gender integration and female participation in science and research, IDIs will be conducted with the administrative and academic staff from the academic and research institutions as shown in table 1 (**Supplimentary material 1**).

2.7. Data collection procedures

Data collection will be conducted over a 12 months period. IDIs will be conducted by a pair of trained research assistants under the supervision of the research team members. Research assistants will be selected from the Master of Public Health (MPH) students in the School of Public Health at University of Zambia. Only students with experience in qualitative research will be selected. Survey questionnaires will be emailed to the respondnets by the members of the research team.

Before data collection, resaerch asistants will undergo training for a total of five days. The training will be conducted by the principal investigator (PIs) and the co-investigator, and will be in two phases: theory and field work. Phase one will include theory in research and will take 3 days. Topics will include: 1) basic principles of qualitative and quantitative research, 2) purpose and objectives of the study, 3) data collection and interviewing techniques, 4) research ethics, and 5) informed consent in human subjects' research. Phase two will be field practical and will include pre-testing the data collection tools and consent form, and will take 2 days, after which the instruments will be revised based on the collected and analysed data and feedback from the research assistants. Each IDI and questionnaire is expected to last between 30 and 45 min.

We plan to conduct record review first followed by IDIs, and then use insights from the literature review and qualitative study to revise and improve the questionnaire for the quantitative survey.

Data collection instruments

For peer review only - http://bmjopen.bmj.com/site/about/guidelines.xhtml

A paper based standard, structured and pre-tested questionnaire (**Supplimentary material 2**) will be used to collect survey data. The questionnaire has been developed based on the existing literature and in consultation with the research team. The questionnaire will also be informed by the findings from the IDIs. In order to to incraese its validity, the questionnaire will be pre-tested and revised during the practical training of data collectors. To make it easirer to administer and measure constructs, questions on attitude an other constructs will be based on a 5 point Likert scale. To make it easy to collect survey data, the questionnaire together with the consent form will be emailed to the selected participants. Instructions will be provided on how to fill in and return the questionnaire.

IDIs will be collected using a pre-tested interview guide (**Supplimentary material 3**) that has been prepared by the research team. The same interview guide will be used for all participants from the study sites. The interview guide will be developed based on specific objectives: a) perspectives on integration of gender in science and research, b) participation/involvement in science and research, c) factors influencing female participation in science and research. The survey questionnaire will also have the same sections but will include an additional section on the sociodemographic and economic characteristics. A checklist (**supplimentary material 4**) will be used for record review. The questionnaire, interview guide and checklist will all be in English. This is because the study participants (academic and research staff) speak English. Before each IDI, respondent sociodemographic data including, age, sex, marital status, occupation, level of education and level of monthly income, will be collected using a short questionnaire. Interviews will be recorded on digital audio-recorders and notes will be taken by research assistants. The data collection tools will be pre-tested and revised before being used in the field. The data collection tools are summarised in table 2 (**Supplimentary material 5**).

2.8. Bias

Possible biases in the study include selection bias, measurement error and confounding due to various background characteristics. To minimize selection bias, systematic stratified sampling will be used to select participants from the linelists maintained in the study sites. In addition, a group of research team members will work together to select the study sample. Other measures to incraese validity of the study will include : a) estimation of the sample size using scientific methods to minimize random error; b) pre-testing and revision of data collection instruments translated into the local languages (Tonga, Bemba and Nyanja) to minimize

For peer review only - http://bmjopen.bmj.com/site/about/guidelines.xhtml

measurement error, c) sampling procedures will be followed to minimize selection bias; d) training of research assistants to minimize measurement error. In addition, research assitants will work under supervison from the field supervisors; e) survey data will be entered and analysed by an independent and competent person to reduce data entry errors; e) coding and analysis of qualitative data will be done by two research team members to increase internal validity. Confounding due to background variables will be controlled for during data analysis. During fieldwork, the team will ensure quality of both quantitative and qualitative data collection process by a) an accompanying note will be attached to each questionnaire to ensure that questionnaires are completely filled in, a) taking extensive notes during key informant interviews, b) comparing notes each day after the interviews.

2.9. Statistical methods and data analysis

Quantitative/survey data will be analysed using SPSS version 21 (IBM, Armonk, NY, USA). Descriptive statistics and frequencies will be used to summarise survey data. Categorical data will be presented using percentages, quantitative data will be presented using the mean and standard deviation (or median and interquartile range). Before running bivariate and multivariate regression analysis, factor analysis will be conducted on each psychosocial measure using principal axis factoring as an extraction method and oblimin rotation. Next, inspection of the scree plot (that is, a plot which displays the eigenvalues associated with a component or factor in descending order versus the number of the component or factor) will be done and sum measures will be created with Eigenvalue score of 1.0 or higher and those items with factor loadings of 0.4 or higher. Reliability test for internal consistency using Cronbach's alpha will be conducted on each of the items. Items that show strong internal consistency (Cronbach's alpha 0.70 or $r > 0.40$) will be combined and averaged into one variable.

Bivariate analysis will be done using Pearson's correlation (for continuous variables) and chi-square (for categorucal variables) to test the association between the independent and outcome variables. Multivariate logistic regression analysis will be conducted to identify predictors of female participation in science and research ($r>0.10$, $p<0.05$).

Qualitative data analysis will be done using an inductive approach which ensures that sub-themes are derived from the pre-determined themes by content-analysis and grouping all similar statements made with respect to particular themes. The voice recordings from the key informant interviews will be transcribed by the research assistants. After verification of accuracy in transcription, each transcript will then be thoroughly read by one research assistant while the other one will be listening to the corresponding voice recording. Each

For peer review only - http://bmjopen.bmj.com/site/about/guidelines.xhtml

transcript will then be compared with the hand-written field notes that the research assistants will prepare during the IDIs. After proof-reading and making corrections, the transcripts will be saved on a password-protected file computer kept by the PI for safety. The Word documents will then be imported into Nvivo 11 MAC for coding and analysis. The exported data will then be coded using a codebook and guiding framework, and the categories and key sub-themes will be identified.

2.10. Patient and public involvement

The study design was determined by the call for application for a research grant on gender dimensions in Science, Technology and innovation under the strategic research fund. Thus, the participants and the public were not directly involved in the conceptualisation and design of the study. Nevertheless, selection of the 16 schools from the two institutions was done in consultation with the authorities from the Ministries of Higher Education and Gender. Further, recruitment of study participants will be done in collaboration with the respective School Deans and their assistants. To inform the institutions about the study, a letter will be written to the registrars and heads of the research institutions. Next, meetings will be held with the two respective School Deans and the heads of the two research institutions to discuss the study and recruitment of survey and IDI participants. Finally, a report will be written and shared with key stakeholders, including the funding organisation and Ministry of Higher Education and Gender. In addition, a dissemination meeting will be held in Lusaka to share the results with the two Ministries and heads of the participating schools and institutions.

Potential risks and mitigation

This study poses minimal risk to study participants. Research assistants will not administer the questionnaire; questionnaires will be delivered to the respondents' office for self-administration. Completed questionnaires will be sealed in an envelope for research assistants to collect. Participation in the study may cause some discomfort from answering certain questions, particularly if the respondent had a negative gender-related experience. To reduce the risk of disclosure of personal or sensitive information, data collectors will be trained to ensure that they advise participants not to share anything that they are not comfortable with; and that they do not have to respond to any question unless they feel comfortable doing so. Since the questionnaire will be self-administered, respondents will have minimal risk of COVID-19 infection.

The study may expose IDI participants and study team members to COVID-19 infection. Bio-safety measures will be taken during data collection including consistent wearing face masks, social distancing and conducting virtual interviews to minimize the risk of infection to the research team and study participants. In addition,

For peer review only - http://bmjopen.bmj.com/site/about/guidelines.xhtml

data collectors and study participants will be encouraged to consistently use face masks and hand sanitisers before and after each interview. Used face masks will be disposed of according to the health guidelines. Data collectors are also trained to minimise any potential discomfort or harm to all participants during all study activities to the greatest extent possible. The study team will reduce any waiting by participants by scheduling appointments during times convenient to participants and interviews are kept to as short a time as possible. To ensure data quality, weekly research meetings will be held to ensure effective and efficient execution of the project.

2.11. Potential benefits

There are no direct individual benefits to participating in the study. If effective, the proposed study findings will be disseminated among the stakeholders and likely to lead to design of evidence informed strategies to improve female participation in science and research.

2.12. Participant confidentiality

Confidentiality of collected data from study participants will be assured throughout the study. The interview will be carried out in participant's office or place of choice. Data collection will not be conducted until we can confirm that the time and location are acceptable to the participants. On completion of IDI and survey, all survey data will be entered in excel and stored on a secure pass-code protected computer kept by the PI. IDI audio recordings will be transcribed and stored on the same computer. Both survey and IDI data will be de-identified and the linking file with identifiable data and basic demographics will be stored in a separate file. Only the PI and some identified research teams will have access to the identifiable data. All analyses will be conducted on de-identified data.

Ethics and dissemination

Ethical clearance and approval were obtained from the University of Zambia Biomedical Research Ethics Committee (UNZA BREC 1674-2022). Permission to conduct the study was granted by the National Health Research Authority. Before being recruited into the study, the purpose, process and duration of the study, benefits and associated risks, privacy and confidentiality will be explained to the respondents. Data collectors will explain that there are no direct individual benefits to participating in the study. They will also explain potential risks the study may pose to study participants and that participation in the study is not likely to pose significant risk. To reduce the risk of disclosure of personal or sensitive information, data collectors will be

For peer review only - http://bmjopen.bmj.com/site/about/guidelines.xhtml

trained to ensure that participants are helped not to share anything that they are not comfortable with; and that they do not have to respond to any question unless they feel comfortable doing so. Participants will also be told that they are free to stop the discussion at any time if they need to. Moreover, participation may expose the study participants and study team to COVID-19 infection. To minimise the risk of infection to the research team and study participants, bio-safety measures will be taken during data collection including social distancing and conducting virtual interviews. In addition, data collectors and study participants will be encouraged to consistently use face masks and hand sanitisers before and after each interview. Used face masks will be disposed of according to the health guidelines. Data collectors will also be trained to minimise to the greatest extent possible any potential discomfort or harm to the participants during all study activities. The study team will minimise any waiting by participants by scheduling appointments during times convenient to participants and ensuring that interviews are kept to as short a time as possible. Those who accept to participate will be asked to sign the consent form (**supplementary material 6**) and be enlisted in the study. Written informed consent will be obtained from each respondent. Those who cannot read or write will be asked to mark with an 'X'. The consent form will be translated into the local language. Participants aged below 18 years will require both parental consent and assent. Privacy of participants and confidentiality of collected data will be assured throughout the study. Interviews will be carried out in participants' private homes or in the participant's place of choice. Data collection will not be conducted until we confirm that the location is acceptable to the participants. To ensure data quality, weekly research meetings will be held to ensure effective and efficient execution of the project. On completion of each data collection phase, all files will be stored on a secure pass-code protected computer kept by the PI. At the end of the data collection process, data will be de-identified and the linking file with identifiable data and basic demographics will be stored in a separate file. Only the PI and some identified research team members will have access to the identifiable data. All analyses will be conducted on de-identified data.

If the study is successful, the findings will be disseminated among the stakeholders. A report will be written at the end of the project and shared with key stakeholders, including the funding organisation and Ministry of Health. In addition, study findings will be disseminated to key stakeholders in Zambia, then through open-access peer reviewed journals, websites and international conferences.

2.13. Limitations

This study has several limitations. First, the study will only include **1,389** survey respondents and 30 IDI participants from four academic and two research institutions. The study findings may not be generalizable

For peer review only - http://bmjopen.bmj.com/site/about/guidelines.xhtml

to other institutions. Moreover, schools connected to the arts and humanities have been excluded as these often have a relatively high proportion of women; the actual proportion of females in these schools is not known. In addition, these schools have been excluded because our project is on Science and Health research. To make the study findings more representative, a large-scale study is needed involving more academic and research institutions. The purpose of the current study is to gain insight on gender integration in science and health research, describe female participation in science and health research and identify the associated factors. Second, non-response may affect the external validity of the study. To mitigate this, we have allowed for a 15% non-response rate. Furthermore, limited access to research data from the selected institutions may affect the quality of the study. Finally, the cross-sectional study will not allow for conclusion on causality. An experimental study is needed to allow for conclusion on causation of low female participation in science and research.

Despite these limitations, we believe that the study is likely going to contribute to the scientific body of knowledge on the integration of gender mainstreaming in science and research. The findings will also provide insight on female participation in science and research and the factors which affect their participation. Currently, no such study has been conducted in the country and information on this subject is scant.

2.14. Costs and payments

There is no cash payment provided to participants for any portion of the study. Participants volunteer the time taken to complete this survey. As a small token of appreciation for their time and opportunity costs, IDI participants will receive a bottle of water or soft drink, in line with UNZABREC procedures.

2.15. Author Contributorship

CS led the design the study, drafting of the study protocol and implementation of the study, and drafted this manuscript. JMZ and MPC contributed to the study protocol. PIS, NBM and NZ contributed to the development of the data collection instruments, enlisting the study sample, and coordinated data collection process. PIS and NZ contributed to the revision of the manuscript. All authors read and approved the manuscript.

2.16. Funding

This work was supported by the National Science and Technology Council (grant number NSTC 101/6/8; **supplementary material 6**), as part of the Gender dimensions in science, technology and innovation under

For peer review only - http://bmjopen.bmj.com/site/about/guidelines.xhtml

For peer review only

the strategic research fund. The funders had no influence on the design, implementation of the study and draft of this manuscript. The protocols underwent a rigorous an external peer review process.

2.17. Conflict of interest

We declare that authors have no conflicting interest

Data sharing statement

Data are available upon reasonable request. Data are available upon reasonable request from the corresponding author and with permission of the UNZA Biomedical Research ethics review board.

References

1. UN Women (1985). World Conference to review and appraise the achievements of the United Nations Decade for Women: Equality, Development and Peace. Available at: http://www.un-documents.net/nflsaw.htm
2. World Conference on Science (1999). Science for the first twenty first Century. Available at: http://www.unesco.org/science/wcs/abstracts/II_4_gender.htm
3. United Nations (2015). Sustainable Development Goals: 17 Goals to transform our world. Available at: https://www.un.org/development/desa/disabilities/envision2030.html
4. ASTI. African Women in Agricultural Research and Development. Available at: https://asti.cgiar.org/gender
5. United Nations Sustainable Development Group (2018). Gender equality and Empowerment of Women: UNCT-SWAP Gender Equality Scorecard. Available at: https://unsdg.un.org/resources/unct-swap-gender-equality-scorecard
6. United Nations (2015). Sustainable Development Goals. Transforming our world: the 2030 Agenda for Sustainable Development. Available at: https://sustainabledevelopment.un.org/post2015/transformingourworld
7. UNESCO 2021. UNESCO Science Report: The race against time for smarter development. Available at: Download the Report | 2021 Science Report (unesco.org)

For peer review only - http://bmjopen.bmj.com/site/about/guidelines.xhtml

8. Gender in Science Innovation, Innovation, Technology and Engineering. Available: https://genderinsite.net/about/who-we-are

9. UNESCO. Sustainable Development Goals for Natural Sciences. Available at: https://en.unesco.org/sustainabledevelopmentgoalsfornaturalsciences

10. European Union, 2022. Science, Research and Innovation Performance of the EU 2022 report: Building a sustainable future in uncertain times. Available at: Science, Research and Innovation performance of the EU 2022 report (europa.eu)

11. UNESCO. Science, Technology and Innovation Policy. Available at: http://www.unesco.org/new/en/natural-sciences/science-technology/sti-systems-and-governance/

12. JICA (2016). Country Gender Profile: Zambia Final Report. Available at: https://www.jica.go.jp/english/our_work/thematic_issues/gender/background/c8h0vm0000anjqj6-att/zambia_2016.pdf

13. Ministry of Gender and Child Development and Central Statistics Office. Gender Status report 2012-2014. Available: https://www.zamstats.gov.zm/phocadownload/Gender/Gender%20Status%20Report%202012-2014%20290616.pdf

14. UNESCO. UNESCO Science report: Towards 2030: Measuring progress towards Sustainable Development Goal 9, available at: https://en.unesco.org/unesco_science_report/sdg9

15. Ministry of Gender and Child Development, 2014. National Gender Policy 2014. Available at: https://genderlinks.org.za/srhrs/zambia-national-gender-policy-2014/

16. Begeny CT, Ryan MK, Moss-Racusin CA, Ravetz G. In some professions, women have become well represented, yet gender bias persists-Perpetuated by those who think it is not happening. Sci Adv. 2020 Jun 26;6(26):eaba7814. doi: 10.1126/sciadv.aba7814.

For peer review only - http://bmjopen.bmj.com/site/about/guidelines.xhtml

17. Cheryan S, Ziegler SA, Montoya AK, Jiang L. Why are some STEM fields more gender balanced than others? Psychol Bull. 2017 Jan;143(1):1-35. doi: 10.1037/bul0000052.
18. National Academies of Sciences, Engineering, and Medicine. Promising Practices for Addressing the Underrepresentation of Women in Science, Engineering, and Medicine: Opening Doors. National Academies Press (US); 2020; PMID: 32369314.
19. Hill, C., Corbett, C., & St Rose, A. Why so few? Women in science, technology, engineering, and mathematics. American Association of University Women, 2010; 1111 Sixteenth Street NW, Washington, DC 20036.
20. Castillo R, Grazzi M, & Tacsir E (2014). Women in Science and Technology. What Does the Literature Say? Inter-American Development Bank. Available at: https://publications.iadb.org/en/women-science-and-technology-what-does-literature-say
21. Lee, H. and Pollitzer, E., 2016. Gender in science and innovation as component of inclusive socioeconomic growth. Portia Limited.19 Nicia, G. and Luisa, S., 2020. A gender-based assessment of Science, Technology and Innovation ecosystem in Mozambique. African Journal of Rural Development, 5(1),
22. Lardies, C.A., Dryding, D. and Logan, C., 2019. Gains and Gaps: Perceptions and Experiences of Gender in Africa
23. Kunda, D., Chembe, C. and Mukupa, G., 2018. Factors that influence Zambian higher education lecturer's attitude towards integrating ICTs in teaching and research. *JOTSE*, *8*(4), pp.360-384
24. UN, 2011. Mainstreaming a gender perspective in science, technology and innovation policy. United Nations Commission on the Status of Women. Available at: Mainstreaming a Gender Perspective in Science, Technology and Innovation Policy (un.org)
25. Nyanja, N. and Musonda, E., 2020. A review of the ICT subject implementation in schools: a perspective of Lusaka Province (Zambia). Education and Information Technologies, 25(2), pp.1109-1127.

For peer review only

For peer review only - http://bmjopen.bmj.com/site/about/guidelines.xhtml

26. Hamusankwa M. and Munsaka, E., Gendered Experiences of Female Engineering Students in Selected Public Universities in Zambia.
27. Kipsoi, E.J., Chang'ach, J.K. and Sang, H.C., 2012. Challenges facing adoption of information communication technology (ICT) in educational management in schools in Kenya. Journal of Sociological research, 3(1), pp.18-28
28. Morsy H (2019). Mainstreaming gender in African policymaking: Ensuring no voice is unheard. Available at: https://reliefweb.int/report/world/mainstreaming-gender-african-policymaking-ensuring-no-voice-unheard
29. Zavale, N.C., 2018. Expansion versus contribution of higher education in Africa: University–industry linkages in Mozambique from companies' perspective. Science and Public Policy, 45(5), pp.645-660
30. United Nations. Envision 2030 Goal 9: Industry, Innovation, and Infrastructure. Available at: https://www.un.org/development/desa/disabilities/envision2030-goal9.html
31. UNESCO. Gender equality guidelines for UNESCO publications. Available at: https://en.unesco.org/system/files/ge_guidelines_for_publications_-_annex_5.pdf
32. UN Women. Gender mainstreaming. Available at: https://www.un.org/womenwatch/osagi/gendermainstreaming.htm
33. UNICEF (2007). Gender main streaming Assessment. Available at: https://www.unicef.org/evaldatabase/files/EO_2007_Gender_Mainstreaming_Report.pdf
34. King N and Horrocks C. 2010. Interviews in Qualitative Research. 1st Edition. SAGE Publications ltd. 25-41.
35. Ary D, Jacobs L, Sorensen C and Walker D. Introduction to research in education. 9th edition. Wadsworth, CA: Cengage Learning, 2013
36. K Massar, C Sialubanje, I Maltagliati, RAC Ruiter. Exploring the perceived effectiveness of applied theater as a maternal health promotion tool in rural Zambia- Qualitative Health Research, 2018; 28(12) 1933–1943
37. Sialubanje C, Sitali DC, Mukumbuta N, Liyali L, Sumbwa PI, Kamboyi HK et al. Perspectives on factors influencing transmission of COVID-19 in Zambia: a qualitative study of health workers and community members. *BMJ Open* 2022;**12:**e057589. doi: 10.1136/bmjopen-2021-057589
38. Sialubanje C, Mukumbuta N, Ng'andu M, Sumani EM, Nkonkomalimba M, Lyatumba DEM et al. Perspectives on the COVID-19 vaccine uptake: a qualitative study of community members and health workers in Zambia. *BMJ Open* 2022;**12:**e058028. doi: 10.1136/bmjopen-2021-058028

For peer review only - http://bmjopen.bmj.com/site/about/guidelines.xhtml

39. Whitley W and Ball J (2002). Statistics review 4: Sample size calculations. Available online: Available online http://ccforum.com/content/6/4/335

40. Jones SR, Carley S, Harrison SM. An introduction to power and sample size estimation. Emerg Med J, 2003;20:453–458

41. Power and sample size calculator. Available at: Sample Size Calculator - Calculate power & sample size for one-sample, two-sample and k-sample experiments (gigacalculator.com)

For peer review only

For peer review only - http://bmjopen.bmj.com/site/about/guidelines.xhtml

Table 1: IDI participant distribution per institution

Participant	UNZA	CBU	Mulungushi	Nkrumah	TDRC	Mt Makulu	Total
Registrar	1	1	0	0	0	0	2
Head of institution	0	0	0	0	1	1	2
Administrative staff	2	2	2	2	0	0	8
Technical staff	0	0	0	2	2	2	4
Lecturers	4	4	2	2	0	0	14
Total	7	7	4	6	3	3	30

For peer review only

Table 2: Summary of data collection techniques

Objective	Technique	Target institution/ group	Instrument
Explore gender integration in science and research	Desk and literature review	UNZA<br>Copperbelt University (CBU)<br>Ministry of Higher Education<br>Ministry of Gender	Checklist
Perspective on factors affecting female participation in science and research	IDI	UNZA<br>CBU<br>Mount Makulu<br>TDRC	Interview guide
Identify the factors that affect female participation in science and research	Survey	UNZA<br>CBU	Questionnaire

For peer review only

INTERVIEW GUIDE FOR ACADEMIC INSTITUTIONS

Gender dimensions and factors affecting female participation in Science and Health Research in Zambia

Theme 1: Policies on Integration of Gender Dimension in scientific and health research

Sub-theme 1.1: Policies on student recruitment

Probe for:

- Available policies on student recruitment in scientific disciplines at undergraduate level
- Available policies on student recruitment in scientific disciplines at postgraduate level
- Dissemination of available policies in the institution
- Extent to which available policies are implemented in the institution
- Policy implementation with regard to promotion of academic staff

Sub-theme 1.2: Policies on staff recruitment and promotion

Probe for:

- Available policies on staff recruitment and promotion in the institution
- Integration of gender in the recruitment and promotion of staff to academic positions
- Integration of gender in the recruitment and promotion of staff to administrative position
- Dissemination of available policies in the institution
- Extent to which available policies are implemented in the institution

Sub-theme 1.3: Policies on Integration of Gender Dimension in Scientific and health research

Probe for:

- Available policies on scientific research and innovation in the institution (eg policy on student recruitment at undergraduate, postgraduate, mention available policies)
- Integration of gender in the available policies
- Mention specific aspects of gender integration in the policies
- Extent to which the available policies are disseminated in the institution
- Extent to which the available policies are implemented in the institution

For peer review only

Theme 2: Participation in Scientific and Health Research

Probe for:

- Extent to which staff in the institution are involved in Scientific and research n
- Extent to which gender dimension is considered in staff participation in scientific and research
- (opportunities for research grants; award of internal and external grants, attending local and international conferences)
- Extent to which gender dimension is considered in staff career path in science and research
- Extent to which gender dimension is considered in staff promotion in scientific and health research
- Suggestions for improving gender integration and female participation in scientific and health research

Theme 3: Factors influencing gender integration and female participation in scientific and health research

Probe for both facilitators and barriers:

- Institutional norms and prejudices on female participation and gender integration in Scientific and health research
- Decision-making on award of research grants in the institution
- Attitude towards female participation in scientific and health research
- Structural factors in the institution (eg, workload, hierarchy)
- Family and personal factors (marriage, religious)

For peer review only

INTERVIEW GUIDE FOR RESAERCH INSTITUTIONS

Exploring the gender dimensions and factors affecting female participation in scientific and health research in Zambia

Theme 1: Policies on Integration of Gender Dimension in scientific and health research

Sub-theme 1.1: Policies on staff recruitment and promotion

Probe for:

- Available policies on staff recruitment and promotion in the institution
- Integration of gender in the recruitment and promotion of staff to scientific and
- Integration of gender in the recruitment and promotion of staff to administrative position
- Dissemination of available policies in the institution
- Extent to which available policies are implemented in the institution

Sub-theme 1.2: Policies on Integration of Gender Dimension in Scientific and health research

Probe for:

- Available policies on scientific research and innovation in the institution (eg policy on recruitment mention available policies)
- Integration of gender in the available policies
- Mention specific aspects of gender integration in the policies
- Extent to which the available policies are disseminated in the institution
- Extent to which the available policies are implemented in the institution

Theme 2: Participation in Scientific and Research

Probe for:

- Extent to which staff in the institution are involved in Scientific research and Innovation
- Extent to which gender dimension is considered in scientific research and Innovation and grant opportunities (award of internal and external grants, attending local and international conferences)
- Extent to which gender dimension is considered in staff career path in Scientific research and Innovation (training opportunities, etc)

For peer review only

- Extent to which gender dimension is considered in staff promotion
- Suggestions for improving gender integration and female participation in the institutions

Theme 3: Factors influencing gender integration and female participation in scientific and health research

Probe for both facilitators and barriers:

- Institutional norms and prejudices on female participation and gender integration in Scientific and health research
- Decision-making on award of research grants in the institution
- Attitude towards female participation in STI in academia and research
- Structural factors in the institution (eg, workload, hierarchy)
- Family and personal factors (marriage, religious)

For peer review only

A. **Research Grants: Local Funding (source within Zambia)**

Name of the School:……………………………………….

Year	2017		2018		2019		2020		2021		Total		Grand Total
	M	F	M	F	M	F	M	F	M	F	M	F	
SDF													
Lecturer 3													
Lecturer 2													
Lecturer 1													
Associate Professor													
Full Professor													
Total for the year													

B. **Research Grants: International Funding (Foreign Source)**

Name of the School:……………………………………….

Year	2017		2018		2019		2020		2021		Total		Grand Total
	M	F	M	F	M	F	M	F	M	F	M	F	
SDF													
Lecturer 3													
Lecturer 2													
Lecturer 1													
Associate Professor													
Full Professor													
Total for the year													

C. **Publications in international Peer Reviewed Journals**

Name of the School:……………………………………….

Year	2017		2018		2019		2020		2021		Total		Grand Total
	M	F	M	F	M	F	M	F	M	F	M	F	
SDF													
Lecturer 3													
Lecturer 2													
Lecturer 1													
Associate Professor													
Full Professor													
Total for the year													

For peer review only

For peer review only

QUESTIONNAIRE FOR ACADEMICIANS & RESEARCHERS

Exploring the gender dimensions and factors affecting female participation in Science and Health Research in Zambia

For peer review only

QUESTIONNAIRE ID...

NAME OF INSTITUTION: ....

SCHOOL: ....

DEPARTMENT: ....

Summary of the study

For peer review only - http://bmjopen.bmj.com/site/about/guidelines.xhtml

Despite the Zambian government making remarkable progress on prioritizing gender mainstreaming, female participation in science, technology and innovation (STI) in academia, research and development is still low. Furthermore, information on the integration of gender dimension and the factors affecting inclusion of men and women in STI in research & development is scant. The aim of this research project is to determine the integration of gender dimensions and the factors that influence female participation in scientific and health research in the country in order to bridge the knowledge gaps and inform policy and programming to support the advancement of gender equality in STI in the country. To this end, the National Science Technology Council has funded this study.

Instructions

You are one of the 400 participants requested to take this questionnaire. Please read carefully and answer all the questions. This questionnaire (60 questions) seeks to collect information on your opinion and perspectives on the extent to which females are involved in and the factors influencing female participation in science-related fields of study in Zambia.

For peer review only - http://bmjopen.bmj.com/site/about/guidelines.xhtml

Section A: Demographic Characteristics

1. Age in years: ……………………………………….

2. Sex:
   1. Male
   2. Female

3. Marital status:
   1. Single
   2. Married
   3. Divorced
   4. Widow
   5. Cohabiting

4. If married, is your partner currently employed?
   1. Yes
   2. No
   3. Not applicable (select this option if not married)

5. If the answer is yes, is your partner working in this organization?
   1. Yes
   2. No

6. Do you have children?
   1. Yes
   2. No

7. If yes, indicate how many (actual number): …………………….

8. Do you have dependants (children or other persons under your responsibility)?
   1. Yes
   2. No

For peer review only - http://bmjopen.bmj.com/site/about/guidelines.xhtml

9. If yes, how many dependants are under your care? Indicate actual number: ……………...

10. Age of dependants:

    1. ___________ between 0 and 5 years
    2. ___________ between 5 and 18 years
    3. ___________ between 19 and 65 years
    4. ___________ 65 years and over

For peer review only

Section B : Educational and Career

11. Indicate your highest academic qualification

    1. Diploma
    2. BSc/BA
    3. MSc/MA
    4. PhD
    5. Postdoc

12. Field of Study (select one most appropriate):

    1. Natural Sciences
    2. Mining
    3. Engineering
    4. Medicine
    5. Veterinary Medicine
    6. Public Health
    7. Health Sciences
    8. Nursing
    9. Others (specify) :…………………

13. Did anyone influence your choice of career?

    1. Yes
    2. No

For peer review only - http://bmjopen.bmj.com/site/about/guidelines.xhtml

14. If yes, who influenced your choice of the career
    1. Personal interest
    2. Father
    3. Mother
    4. Sibling
    5. Family member
    6. Friend
    7. Teacher
    8. Career counsellor
    9. Others (specify)………………………………………………..

15. Why did you enroll in this program initially?
    1. Interest in the subject matter
    2. Field in which I excelled
    3. Influence of faculty members
    4. Influence of advisor or mentor
    5. Prospects for personal development
    6. Social aspect of expected job
    7. Work conditions and benefits expected
    8. I always thought I would study in this field
    9. Other, please specify:………………..

Section C: Employment

16. Employment status:
    1. Permanent and Pensionable
    2. Contractual

17. Indicate the number of years working in this institution…............

18. Indicate your current position:
    1. Staff development fellow;

For peer review only - http://bmjopen.bmj.com/site/about/guidelines.xhtml

2. Lecturer 1
3. Lecturer 2
4. Lecturer 3
5. Associate Professor Researcher
6. Full Professor
7. Administrative staff

19. Indicate the number of years in this position…..................

20. From your personal point of view, which one of the following factors were important in obtaining your current position?
    1. Earlier cooperation with faculty members of your current work place
    2. Personal contacts at your current work place previous to obtaining your position
    3. A network of people who could testify to your professional qualifications
    4. Programs promoting gender parity at work
    5. Expansion of higher education sector
    6. Formal or informal professional experience outside the academic sector
    7. Number of publications or patents
    8. Number of grants awarded
    9. Citation impact of prior work
    10. Value of grants awarded

For peer review only

21. To what extent are you satisfied by your current job? Please rate your satisfaction using the scale below

1	2	3	4	5
Very unsatisfactory	Unsatisfactory	I don't know	Satisfactory	Very satisfactory

Section B: Academic Research History

22. Have you been involved in academic research?

For peer review only - http://bmjopen.bmj.com/site/about/guidelines.xhtml

1. Yes
2. No

If the answer is No, indicate "Not applicable" on questions 20 to 22, and proceed to question 23.

23. If the answer is yes, at what level have you been involved?
    1. Research Assistant
    2. Field Supervisor
    3. Collaborator
    4. Co-investigator
    5. Principal investigator
    6. Not applicable

24. If the answer is yes, indicate number of research projects in past 2 years:
    1. 1-2
    2. 2-3
    3. 3-4
    4. 5-6
    5. >6
    6. Not applicable

25. Indicate reasons for involvement in research:
    1. Requirement for certification
    2. Academic promotion
    3. Contribution to knowledge
    4. For international recognition
    5. Not applicable
    6. Other (specify)………

26. If the answer is no, indicate the reason:
    1. Limited skills in research;
    2. Limited access to research funds;
    3. Restrictive work policy;

For peer review only - http://bmjopen.bmj.com/site/about/guidelines.xhtml

4. Not interested in research;
5. Other (specify)…………

27. Are you currently seeking a new job?
    1. Yes
    2. No

28. If yes, why?
    1. Better salary
    2. Closer to family
    3. Better work environment
    4. More suitable job to my training & experience
    5. Other (specify)……………..

29. Is this your first job in your field?
    1. Yes
    2. No

30. Do you consider yourself to have enough opportunities to participate in research projects?
    1. Yes
    2. No

31. If yes, mention which ones
    1. Locally funded research projects
    2. Internationally funded research projects
    3. Both locally and internationally funded research projects
    4. Not applicable

32. If the answer is no, mention why?
    1. Did not receive any funding
    2. Did not receive any information on funding opportunities
    3. Could not find information on funding opportunities
    4. Was not authorized to apply for research funds
    5. Did not look into how to access to funding opportunities

For peer review only - http://bmjopen.bmj.com/site/about/guidelines.xhtml

6. Other (specify).......

33. Do you consider yourself to have enough opportunities to participate in conferences and workshops?
    1. Yes
    2. No

34. If yes, mention which ones?
    1. Local conferences and workshops
    2. International conferences and workshops
    3. Both local and international conferences and workshops
    4. Not applicable (***select this option if the response to question 30 is "No***")

35. If the answer is no, mention why?
    1. Did not receive any funding
    2. Did not receive any information on opportunities
    3. Could not find information on opportunity
    4. Was not authorized
    5. Did not look into how to access such opportunities
    6. Other, specify.......

36. How frequently do you engage in presentation to the public at a scientific conference
    1. Never
    2. Once per year
    3. Twice per year
    4. Four times per year
    5. More than four times per year

37. Have you ever had a period of time during your professional training or in your career where it was difficult for you to fulfill your professional responsibilities because of your personal responsibilities?
    1. Yes
    2. No

For peer review only - http://bmjopen.bmj.com/site/about/guidelines.xhtml

38. If yes, what were the main reasons?
    1. Care for children
    2. Care for family member (other than children)
    3. Illness/accident
    4. Burn out
    5. Depression
    6. Change in a marital situation (for example divorce)
    7. Death in family or of closely related person
    8. Other, specify……………
    9. Not applicable (**select this option if your answer to question 42 is "No**")

39. Have you ever received any awards for your accomplishments?
    1. Yes
    2. NO

40. Have you ever been invited to participate in committees by the Ministry responsible for Science, Technology and Innovation (STI) in Zambia
    1. Yes
    2. No

41. Have you applied for a research grants before?
    1. Yes
    2. No

42. If yes, how many times have you applied for grants from national and international institutions over the past 2 years? Indicate number:……………..

43. If yes, what was your role?
    1. Principal investigator
    2. Part of a team

44. Where did you apply?
    1. National funding institution
    2. International funding institution

For peer review only - http://bmjopen.bmj.com/site/about/guidelines.xhtml

3. Both national and international funding institution

45. How many national grants have you won? Indicate number and amount……………………...

46. How many international grants have you won? Indicate number and amount………..……….

Gender equality in research

47. In your opinion, can female researchers be as successful in winning research grants as their male folks?
    1. Yes
    2. No
    3. I don't know

48. In your opinion, can female researchers be as successful in publishing their scientific work as their male folks?
    1. Yes
    2. No
    3. I don't know

49. Do you think the field of science and research is equally suited for both men and women?
    1. More suitable for men
    2. More suitable for women
    3. Suitable for both men and women

50. Are you aware of instances of a pay gap between men and women who have the same responsibilities in your organization?
    1. Yes
    2. No

Harassment at a place of work

For peer review only

For peer review only - http://bmjopen.bmj.com/site/about/guidelines.xhtml

Harassment can take any form such as verbal, physical, psychological, sexual or other forms of abuse by a colleague, superior of the same or opposite sex. It can also include being denied access to certain services or privileges on account of sex, place of origin, ethnicity race and other stereotypes

51. Have you had any experience of harassment at your current or a former workplace?
    1. Yes I have witnessed it
    2. Yes I have heard second hand reports
    3. No
    4. I can't tell

52. If yes, what form of harassment did you experience?
    1. Verbal abuse from a colleague
    2. Verbal abuse by a superior
    3. Denied access to research funds
    4. Sexual abuse
    5. Others, Specify…………

53. Have you ever left a job on account of harassment?
    1. Yes
    2. No

54. If yes, to what extent did harassment at work contribute to your leaving the job?
    1. It was a contributing factor
    2. It was not a factor
    3. Not applicable
    4. I have not left

THE END

For peer review only - http://bmjopen.bmj.com/site/about/guidelines.xhtml

Informed Consent Form

Exploring the gender dimensions and factors affecting female participation in Science, Technology and Innovation (STI) in Academia, Industry-Research & Development in Zambia

Background

Hello, my name is ___________________________ I am working with the Levy Mwanawasa Medical University and University of Zambia. I am part of the research team on "Exploring the gender dimensions and factors affecting female participation in Science, Technology and Innovation (STI) in Academia, Industry-Research & Development in Zambia". Despite the Zambian government making remarkable progress on prioritizing gender mainstreaming, female participation in science, innovation, research and development is still low. Moreover, information on the integration of gender dimension and the factors affecting inclusion of men and women in Science, innovation, academia, research & development is scant. To this end, the National Science Technology Council has funded this study.

Purpose: The aim of this research project is to explore the integration of gender dimensions and the factors that influence female participation in STI in academia-industry-research & development in the country in order to bridge the knowledge gaps and inform policy and programming to support the advancement of gender equality in STI in the country. We would like to speak with you because you are …………….. We would like to understand your opinion and perspectives on the extent to which females are involved in and the factors influencing female participation in science-related fields of study in Zambia.

Process: You will be one of approximately 450 respondents asked to participate in this discussion. We would like to ask you some questions in order to understand your opinions and perspectives on this subject. The discussion should take no longer than 60 minutes. To ensure that I don't miss or forget anything during the discussion, your responses to the various questions will be recorded using a digital recorder. We will not write your name, instead we will ask you to sign on this form; we will keep your signature private. Responses from the various questions will be summarised, analysed and a report written. When we write reports from this discussion, we will not show what you said nor are we going to use your name or signature at any time.

Potential Benefits: You will receive no direct benefit from your participation in this discussion. However, your participation may help the Zambian government to understand the integration of gender dimensions and the factors that influence female participation in STI in academia-industry-research & development in the country. This information will help to bridge the knowledge gaps and in turn, inform policy and programming to support the advancement of gender equality in STI in the country.

Risks and Discomforts: The risks of taking part in this study are that other people will hear your responses. It is important that you do not share anything that you are not comfortable with. If you or someone in your family had a bad experience, it may be difficult or uncomfortable to remember or share it. You do not have to respond to any question unless you feel comfortable doing so. You are free to stop the discussion at any time if you need to.

.

Alternatives: You can choose not to take part in this discussion. If you decide not to take part or withdraw from this discussion, you will not suffer any penalty or lose any benefits to which you are entitled.

Participant Costs and Payments: You will not be paid to participate in this discussion. There are no costs to you for participating.

Participant's Rights: By agreeing to participate in this discussion, you do not waive any of your legal rights. Giving consent means that you have heard or read the information about this discussion and that you agree to participate. You will be given a copy of this form to keep. If you have questions or concerns at any time, you can contact the Principal Investigator, Dr. Sialubanje Cephas on +260-977-441 273, the Co-Investigator, Prof Joseph Mumba Zulu on +260 971 591 388 or any of the staff from the University of Zambia Biomedical Research Ethics Committee on +260-21-1-256067.

Right to Refuse or Withdraw: Taking part in this discussion is voluntary. You have the right to refuse to take part. If you decide

For peer review only - http://bmjopen.bmj.com/site/about/guidelines.xhtml

to be in this discussion, and then change your mind, you can withdraw from it at any time and to skip questions you may deem personal or otherwise without any repercussions. Your participation is voluntary. If you choose to take part, you have the right to stop the discussion at any time.

Confidentiality: The results of this study will be kept strictly confidential, and used only for research purposes. Your identity will be concealed in as far as the law allows. Your name will not appear anywhere on the coded forms with the information. Paper and computer records will be kept under lock and key and with password protection respectively. The interviewer has discussed this information with me and offered to answer my questions. For any further questions, I may contact the Chairperson, UNZABREC on the following details___________________

STATEMENT OF CONSENT/ASSENT

.... has described to me what is going to be done, the risks, the benefits involved and my rights regarding this study. I understand that my decision to participate in this study will not alter my usual medical care. In the use of this information, my identity will be concealed. I am aware that I may withdraw at anytime. I understand that by signing this form, I do not waive any of my legal rights but merely indicate that I have been informed about the research study in which I am voluntarily agreeing to participate. A copy of this form will be provided to me.

Name:…………………………Signature of participant ……………………..Age..…………….
Date (DD/MM/YY)……………………………

Name of Witness……………………………….. Signature of Witness…………………………
Date (DD/MM/YY)…………………………………………….

Name…………………………Signature of parent or guardian for minors ……………………
Date(DD/MM/YY)…………………………

Name……………………………………Signature of Interviewer …………………………Date (DD/MM/YY)………………………...

If you have any further questions please contact the University of Zambia Biomedical Research Ethics Committee

Telephone: 256067 Ridgeway Campus

Telegrams: UNZA, LUSAKA P.O. Box 50110

Telex: UNZALU ZA 44370 Lusaka, Zambia

Fax: + 260-1-250753

E-mail: unzarec@unza.zm

Assurance No. FWA00000338 **IRB00001131 of IOR G0000774**

By signing below you are agreeing to participate in the discussion which indicates that you have read this consent form (or have had it read to you), that your questions have been answered to your satisfaction, and that you voluntarily agree to participate in this research study. You may keep a copy of this for your records.

Signature or thumb print: ____

Date: ____

Signature of Impartial Witness: ______________________________________

Date: ____

For peer review only - http://bmjopen.bmj.com/site/about/guidelines.xhtml

For peer review only

For peer review only - http://bmjopen.bmj.com/site/about/guidelines.xhtml

STROBE Statement—Checklist of items that should be included in reports of cohort studies

**Item**	**Recommendation**	Page
Title and abstract	Indicate the study's design with a commonly used term in the title or the abstract	1
	Provide in the abstract an informative and balanced summary of what was done and what was found	2
Introduction		
Background/rationale	Explain the scientific background and rationale for the investigation being reported	4
Objectives	State specific objectives, including any pre-specified hypotheses	5
Methods		
Study design	Present key elements of study design early in the paper	6
Setting	Describe the setting, locations, and relevant dates, including periods of recruitment, exposure, follow-up, and data collection	6
Participants	Give the eligibility criteria, and the sources and methods of selection of participants. Describe methods of follow-up	7
Study size	Explain how the study size was arrived at	8
Variables	Clearly define all outcomes, exposures, predictors, potential confounders, and effect modifiers. Give diagnostic criteria, if applicable	9
Data sources/ measurement	For each variable of interest, give sources of data and details of methods of assessment (measurement). Describe comparability of assessment methods if there is more than one group	9
Bias	Describe any efforts to address potential sources of bias	11
Statistical methods	Describe all statistical methods, including those used to control for confounding	12
	If applicable, explain how loss to follow-up was addressed	
Patient and public involvement		12
Ethical considerations		13
Limitations		14
Funding		15
